# Seasonal and elevational changes of plant‐pollinator interaction networks in East African mountains

**DOI:** 10.1002/ece3.10060

**Published:** 2023-05-11

**Authors:** Fairo F. Dzekashu, Christian W. W. Pirk, Abdullahi A. Yusuf, Alice Classen, Nkoba Kiatoko, Ingolf Steffan‐Dewenter, Marcell K. Peters, H. Michael G. Lattorff

**Affiliations:** ^1^ International Centre of Insect Physiology and Ecology (icipe) Nairobi Kenya; ^2^ Social Insects Research Group, Department of Zoology and Entomology University of Pretoria Pretoria South Africa; ^3^ Department of Animal Ecology and Tropical Biology, Biocenter University of Würzburg Würzburg Germany; ^4^ Present address: Department of Chemistry University of Nairobi Nairobi Kenya

**Keywords:** bees, biodiversity, climate change, Eastern Afromontane Biodiversity Hotspot, ecosystem services, elevation gradients, insect conservation, network robustness, pollination, tropical mountains

## Abstract

Across an elevation gradient, several biotic and abiotic factors influence community assemblages of interacting species leading to a shift in species distribution, functioning, and ultimately topologies of species interaction networks. However, empirical studies of climate‐driven seasonal and elevational changes in plant‐pollinator networks are rare, particularly in tropical ecosystems. Eastern Afromontane Biodiversity Hotspots in Kenya, East Africa. We recorded plant‐bee interactions at 50 study sites between 515 and 2600 m asl for a full year, following all four major seasons in this region. We analysed elevational and seasonal network patterns using generalised additive models (GAMs) and quantified the influence of climate, floral resource availability, and bee diversity on network structures using a multimodel inference framework. We recorded 16,741 interactions among 186 bee and 314 plant species of which a majority involved interactions with honeybees. We found that nestedness and bee species specialisation of plant‐bee interaction networks increased with elevation and that the relationships were consistent in the cold‐dry and warm‐wet seasons respectively. Link rewiring increased in the warm‐wet season with elevation but remained indifferent in the cold‐dry seasons. Conversely, network modularity and plant species were more specialised at lower elevations during both the cold‐dry and warm‐wet seasons, with higher values observed during the warm‐wet seasons. We found flower and bee species diversity and abundance rather than direct effects of climate variables to best predict modularity, specialisation, and link rewiring in plant‐bee‐interaction networks. This study highlights changes in network architectures with elevation suggesting a potential sensitivity of plant‐bee interactions with climate warming and changes in rainfall patterns along the elevation gradients of the Eastern Afromontane Biodiversity Hotspot.

## Graphical abstract

We recorded plant‐bee interactions at 50 study sites in Kenya between 515 and 2,600 m asl for a full year, following four major seasons. We analysed elevational and seasonal network patterns. We found strong elevational and seasonality patterns in network structures, which were attributed to seasonal changes in climate, floral resource availability, and bee assemblages.

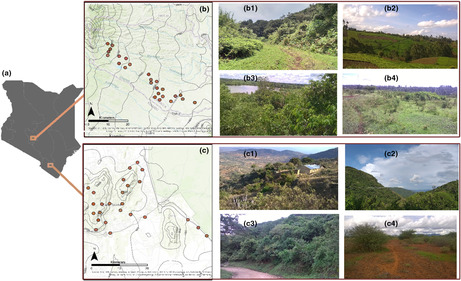

## 1 INTRODUCTION

The mutualistic interactions between plants and pollinators are among the most highly regarded foci in ecology, providing valuable insights into biotic network structures as a basis of species co‐existence, diversification, and ecosystem functioning (Fortuna & Bascompte, 2006; Tylianakis et al., 2010). In the past two decades, research on plant‐pollinator interactions has been characterized as nested (Almeida‐Neto et al., 2008), specialized (Blüthgen et al., 2007), and modular (Olesen et al., 2007), and to be constrained by functional trait matching between flowers and pollinators (Albrecht et al., 2018; Garibaldi et al., 2015). However, currently little is understood on how climatic gradients and prospective climate warming will change plant‐pollinator interaction networks in the future (Hoiss et al., 2015). Much of the knowledge on the change in network metrics along broad climatic gradients was gained in meta‐analyses, summarizing network data that differed in taxonomic resolution (single taxa vs multi‐taxa interactions), sampling effort, biogeographic context, and/or season (Sargent & Ackerly, 2008; Schleuning et al., 2012; Schwarz et al., 2020; Vizentin‐Bugoni et al., 2018). However, as plant‐pollinator interactions turned out to be highly dynamic in space and time (Baldock et al., 2011; Petanidou et al., 2008), and as different pollinator groups naturally differ substantially in their foraging patterns (Mertens et al., 2018, 2020), such meta‐analyses are limited to finally sharpen our understanding about the main drivers and mechanisms underlying network architectures. A mechanistic understanding, however, is gaining in importance, given the speed of global change and the observed decline of fundamental ecosystem functions, including pollination (Dainese et al., 2019; Powney et al., 2019), threatening food production and human wellbeing (Hass et al., 2018; Martins et al., 2021).

Studying species communities with standardized sampling methods along elevation gradients is a powerful tool to investigate major drivers of biodiversity (Classen et al., 2020; Hoiss et al., 2015). Along elevational gradients, abiotic and biotic factors, such as climate or resource availability change over very small spatial scales (Körner, 2007), which can fundamentally restructure species communities (Sponsler et al., 2022). However, while many studies have centered on the impact of elevation on abundance and species richness of individual taxonomic or trophic groups (e.g., plants or pollinators) (Dzekashu et al., 2022; Maicher et al., 2018; Peters et al., 2016), there still remains an apparent dearth of studies addressing patterns and drivers of species interaction networks with elevation (Hoiss et al., 2015; Maunsell et al., 2015), especially on tropical mountains (Classen et al., 2020; Mertens et al., 2021; Ramos‐Jiliberto et al., 2010).

Bees are important contributors to the conservation of wild plant diversity (through pollination services), maintenance of ecosystem stability, and functioning of natural habitats (Dainese et al., 2019; Potts et al., 2016). They are very sensitive to changing climate and food resources, as such their population dynamics are highly structured by the level of resource availability in a given area (Crone & Williams, 2016; Vaudo et al., 2015).

The impact of elevation and seasonal changes in climate (Dzekashu et al., 2022) on plant‐pollinator interaction networks still remains elusive for tropical elevation gradients. Seasonality substantially influences plant phenology and animal physiology at both high and low latitudes (Thuiller, 2007). Here, the ambient conditions associated with tropical elevations throughout the year can foster specialization along the climate niche axis and high turnover of species in space (elevation) and time (with seasonal changes) (Maicher et al., 2020; Schmitt et al., 2021). The climate‐related temporal distribution of flowering plants along elevational gradients can lead to the seasonal apportioning of visitation (Baldock et al., 2011). Hence, flowering plant species in natural communities across an elevation gradient having similar traits with overlapping phenology might facilitate higher visitation rates when flowering simultaneously (de Santiago‐Hernández et al., 2019). Also, since temperature correlates with developmental periods, environments of higher temperature may facilitate earlier emergence, more generations per season, and elevational dispersal patterns to optimize the use of seasonal fluctuating resources (Hegland et al., 2009). Nonetheless, empirical evidence of climate‐driven seasonal plant‐pollinator network dynamics is mainly unknown, particularly across tropical environmental gradients.

Increased specialization at elevations with a favorable climate can lead to increased competition for available floral resources among co‐occurring species, portentous of a stricter co‐evolutionary relationship between the interacting species (Sebastián‐González et al., 2015; but see Schleuning et al., 2012). Due to narrower niche‐breadths, species in the tropics form more specialized interactions, resulting in assemblages with more aggregated species than temperate organisms (Vázquez & Stevens, 2004). Even though under such circumstances, we would expect specialization to decrease in areas with reduced floral resources and reduced temporal windows of bee activities, it remains unclear as to how changing climate and turnover of bees and plant flowering resources would shape specialization patterns across tropical elevations. Owing to reduced preferred feeding resources at higher elevations, bees with specialized feeding requirements shift their foraging to areas with sufficient varieties of feeding resources and suitable climate, thus leaving the set of generalist feeders unaltered at higher elevations (Tylianakis et al., 2010). We therefore predict that nestedness will increase along the elevation gradient. High species richness is also known to promote lower connectance (Jordano, 2016), whereas connectance is often linked to ecosystem stability. Therefore, as specialization reduces across elevation, we expect connectance (and thus stability) and increased generalization (Tylianakis et al., 2010) to increase across elevation where abiotic (e.g., temperature) and biotic factors (available feeding resources) often limit species richness, hence interaction networks (Jordano, 2016). Nonetheless, there still exists a dearth in knowledge towards understanding how such patterns are organized in a contemporized climate scenario along tropical mountains. Highly rewired network systems mirror the capacity of one trophic level to buffer extinction events in another trophic level. They strengthen resilience (Gresty et al., 2018) by limiting the risk of species loss due to a greater ability for species to switch interactions as a response. Plants flowering phenology and pollinator emergence as a result of favorable climatic conditions might strongly contribute to interaction rewiring (Schwarz et al., 2020). However, pragmatic studies highlighting the drivers of network link rewiring patterns on tropical mountains are still lacking. Modularity increases with species richness (Vizentin‐bugoni et al., 2018), and therefore becomes pronouncedly high in tropical networks, more so at lower elevations, whereby species‐rich communities often lead to ideal niche‐partitioning, hence strong modularity (Martín González et al., 2010; Olesen et al., 2007). We therefore would expect modularity to decrease with increasing elevation. However, there is limited knowledge on how ecological drivers might constrain subsets of species (subcommunities) from interacting more among themselves.

In this study, we analyzed the architecture of plant‐pollinator networks across an elevation gradient in the Eastern Afromontane Biodiversity Hotspots (EABH) in Kenya, East Africa, and disentangled the underlying drivers shaping the observed patterns. Specifically, we asked the following questions: (1) How do plant‐bee species interaction networks change with elevation in the EABH? (2) How does the topology of networks change across different seasons? and (3) What drives the observed patterns in these network structures along this tropical elevation gradient?

## 2 MATERIALS AND METHODS

### 2.1 Study area and time of study

The study was conducted along two elevational gradients of the Eastern Afromontane Biodiversity Hotspot (EABH) in Kenya: Taita Hills (38°10′ to 39°03′ E, −3°15′ to −4°0´S) from 525 to 1865 m asl and Murang'a in the central region of Kenya (36°43′ to 37°27′ E, 0°34′ to 1°5´S) from 1470 to 2530 m for a whole year covering the four phenological timescales in this region (i.e., July: long dry and cold season; September–October: short dry and cold season; November and December: short‐rainy and warm season; March–April: long rainy and warm season) between June 2019 and May 2020.

The lowlands are characterized by a sub‐tropical climate that is highly arid throughout most parts of the year (especially during the long dry season) giving rise to savannah vegetation, while the highlands are covered with montane forests closely bordered by intensive agricultural lands and human settlements. Farming and grazing activities are equally carried out and several honeybee hives are deployed by local indigenes into the forested highlands. Along the elevation gradient, the landscape is made up of savannah, shrublands, indigenous bushlands, pasture, and human settlement with some subsistence farming activities (Dzekashu et al., 2022). Seasonality in climate is pronounced in the study region. Here, rainfall displays a bimodal seasonal configuration with a short‐rainy interval between November and December, ensued by a dry interval of 2–3 months, whereas prolonged heavy rainfalls are typical from March to May followed by a long dry interval of 5 months. Mean monthly precipitation amount ranges between ~25 and 85 mm during the cold‐dry seasons and ~ 95 to 160 mm during the warm‐wet seasons from low to highlands (Figure 5e), while mean monthly air temperature ranges from ~28.6°C to 29.7°C during the cold‐dry seasons and ~28.9°C to 30°C during the warm‐wet seasons from highlands to the lowlands across the elevation gradients of this region (Figure 5f).

**FIGURE 1:**
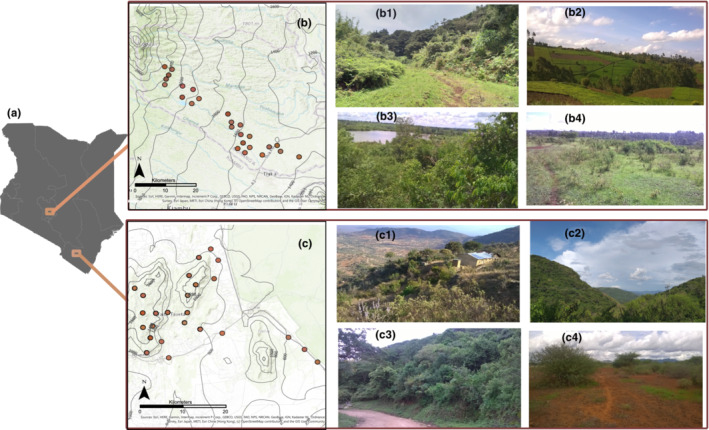
Map of the two elevation gradients in Kenya (a), Murang'a (b), and Taita Hills (c). Study plots (brown dots) are geographically positioned along elevation gradients. Each gradient contained 25 study plots. Contour lines in b and c indicate elevation levels. Photos in b1–b4 exemplify the vegetation structure around study plots along elevation gradients in Murang'a (b1 = 2414 m asl, b2 = 2035 m asl, b3 = 1530 m asl, b4 = 1462 m asl), while photos in c1–c4 show the vegetation structure around study plots along elevation gradients in the Taita Hills (c1 = 1624 m asl, c2 = 1344 m asl, c3 = 981 m asl, c4 = 526 m asl). All photos were taken during sampling events.

Twenty‐five plots of 100 m × 100 m were selected along each of the two studied elevational gradients making a total of 50 plots within the study area (Figure 1). Plots within the forests were positioned in less dense areas and or regrowth vegetation with easy access. The distance between individual plots ranged from 2.3 to 8.2 km (larger than the estimated foraging range of most tropical bee species, Greenleaf et al., 2007; Wikelski et al., 2010) and succeeding elevation increments of ca. 100–250 m between adjoining plots (Figure 1, Table S2).

### 2.2 Plant‐bee interactions

Interaction observations were conducted by the same team of three experienced observers throughout the entire study to avoid biases. Sampling of species interactions was performed on each plot in each of the four major seasons for 2 h by all three observers and restricted to a time between 09 and 17 h (local time). This time range is considered to be the period of peak activities of some tropical bee species (Oliveira et al., 2021). We exclusively conducted sampling in time periods without rain and without heavy winds or fog. We followed a slow, gentle, and parallel movement around an entire plot during sampling, observing flowers for potential bee visitors (Westphal et al., 2008). An interaction (observation) was defined when a bee touched the sexual parts of a flower (anthers and/or stigma). We collected all bees visiting flowers using standardized sweep nets and an improved Prokopack aspirator. The aspirator (Model 1419, John Whock) was used to collect bees visiting tall plants that were unreachable or for which sweep netting was difficult. This device made it possible to collect bees from trees up to ~4 m in height. In addition, samples of all visited plants were collected and high‐resolution plant photos were made using digital cameras (Canon EOS Rebel T7 DSLR and Samsung Galaxy J8 mobile phone) for later identification. All sampling observations were standardized and piloted during the four seasons described above. All bees were identified to genus level and later sorted to either species or morphospecies level with the help of an expert (Jayne Macharia) from the National Museums of Kenya (NMK) following Michener (2007) and Eardley et al. (2010). All plant species were also identified at the species level by a plant taxonomist (Kennedy Matheka) from the NMK.

### 2.3 Climate data

Climatic variables were obtained from the Climatologies at high resolution for the Earth's Land Surface Area (CHELSA) database (Karger et al., 2017), providing climate data at a 30 arc‐seconds (ca. ~1 km^2^) resolution for each of our study plots. The following monthly time series climate variables were extracted and the mean values for the five most recent years (2015–2020) including the periods of our sampling events calculated: tas: mean daily air temperature for each month (MMT) across the different seasons, that is tas_06, tas_09, tas_10 (cold‐dry seasons) and tas_03, tas_04, tas_11, tas_12 (warm‐wet seasons), and pr: monthly precipitation amount (MMP) across the different seasons, that is pr_06, pr_09, pr_10 (cold‐dry seasons) and pr_03, pr_04, pr_11, pr_12 (warm‐wet seasons) (Table S1). This database is extensively used in ecological studies as it offers more accurate precipitation data across elevations than other databases (e.g., Marcondes et al., 2020; Pironon et al., 2019).

### 2.4 Network indices

Since we recorded actual visitation frequencies by bee species on different plant species, we estimated all network indices using quantitative data (weighted networks), which are considered finest with regard to information content and precision (Blüthgen, 2010; Dormann & Strauss, 2014). All network indices were calculated using the “bipartite” package (Dormann et al., 2017) in the R statistics platform version 4.0.3 (R Core Team, 2020). We choose the following indices:

#### 2.4.1 Weighted nestedness overlap and decreasing fills (*w*NODF)

It describes a nonrandom pattern where links of specialist species tend to interact with generalist species. The values of nestedness ranged from 0 to 100, where 0 indicates fully nested networks (i.e., low proclivity of specialists to interact with generalists, with less interacting propensity among each other, Classen et al., 2020) and 100 represents random networks (i.e., specialists are inclined to interact with generalists, which in turn interact more among each other, Almeida‐Neto & Ulrich, 2011; Petanidou et al., 2018). Weighted nestedness was calculated using the *weighted NODF* algorithm.

#### 2.4.2 Specialization (*H2*′ and *d*′)

We calculated two measures of specialization: the degree of network specialization (*H*2′) and the degree of species specialization (*d*′) (Blüthgen et al., 2006). *H*2′ characterizes the average degree of specialization between species in the entire network, describing the complementarity of interactions. The degree of interaction specialization (*d*′) estimates the strength in the specialization of interaction networks at the species level (focal species) by quantifying the deviation of actual interactions from a null model, thereby adequately quantifying variations within a network (Blüthgen et al., 2006; Miranda et al., 2019). The values of specialization range from 0 to 1, with higher values indicating higher specialization and lower values the inverse. *H*2′ was calculated using the *H2fun* algorithm, while the species‐level specialization *d*′ was calculated for each species using the *dfun* algorithm and later averaged across species for each plot or for each seasonal sample on each plot.

#### 2.4.3 Weighted connectance (*w*C)

Often considered as interaction diversity (Tylianakis et al., 2010), it is the sum of all realized links (density) in a network divided by the possible links (Bersier et al., 2002). Connectance is used to estimate community complexity and to detect stability in the ecosystem (Russo & Shea, 2017; Tylianakis et al., 2010). Its values range between 0 and 1, with higher values indicating increases in realized interactions. Connectance is known to decrease with species richness (Vizentin‐bugoni et al., 2018), whereas increased connectance is equivalent to increased generalization. We used the *weighted connectance* algorithm to obtain our weighted connectance matrix.

#### 2.4.4 Modularity (*Q*)

Here, subsets of species (link‐rich clusters or subcommunities) interact more among themselves as compared to other species in the network forming a module or compartment (Dehling, 2018). Modularity increases the stability in plant‐pollinator networks by buffering the effects of perturbations across link‐rich clusters (Carstensen et al., 2016; Tylianakis et al., 2010; Zanata et al., 2017). The values in modularity range from 0 (no link‐rich clusters) to 1 (total compartmentalization of species). We estimate the modularity parameter for each plot and season using the *ComputeModules* algorithm (Dormann et al., 2021).

#### 2.4.5 Link rewiring (*β*_OS_)

It quantifies interaction reassembly or temporal dynamics between seasonal networks (i.e., dissimilarity due to shared species subwebs). This rewiring can be attributed to variations of interacting subwebs (Poisot et al., 2012; Schwarz et al., 2020). The values for *β*_OS_ range from 0 to 1; where higher values indicate higher rewired link dissimilarity or increased variation in seasonal interacting subsets (i.e., a gain in seasonal reassembled interactions, CaraDonna et al., 2017). We used the *betalinkr* function (Dormann et al., 2022; Schwarz et al., 2020) to estimate the degree of interaction dissimilarity between seasonal networks (i.e., between the long dry‐cold & short dry‐cold for the cold‐dry season, and short wet‐warm & long wet‐warm for the warm‐wet season). Because of too few or no interactions recorded in some plots during sampling events across the different seasons, and in order to obtain the best representative and comparative seasonal reassembled interaction networks, we reduced the number of plots for the link dissimilarity analysis to only include seasonal‐paired plots where interactions were recorded during the cold‐dry and warm‐wet seasons, respectively.

Moreover, we did not correct the effects of network size before determining our network properties since we considered network size to be a very important factor that is strongly dependent on seasonal network composition (e.g., Schwarz et al., 2020). However, we still adjusted for the effect of network size by first calculating its seasonality pattern across elevation and then included species diversity measures as predictor variables in our multivariate analysis (e.g., Schwarz et al., 2020).

### 2.5 Statistical analyses

All statistical analyses were performed using the following packages: “MuMIn” (Barton, 2009), “Vegan” (Oksanen et al., 2014) “mgcv” (Wood, 2006), and “corrplot” (Wei et al., 2017) in the R statistics platform version 4.0.3 (R Core Team, 2020). Network indices were calculated for each plot and at different levels: cross‐season data sets split up into cold‐dry (1) and warm‐wet season (2). Since the data collection procedure was harmonized across seasons for each plot, seasonal data were pooled together (i.e., long dry‐cold & short dry‐cold as cold‐dry season; short wet‐warm & long wet‐warm as warm‐wet season) to increase the sample size for individual networks, enhance fitting of extreme values and reduce bias and uncertainties.

To determine patterns of network assemblages across elevation gradients, we used generalized additive models (GAM). In general additive models, the relationship between regressands and regressors is unconditional to specific functions (Peters et al., 2019). As such, GAM uses nonparametric smoothers to suffuse simple and complex nonlinear and linear relationships (Wood, 2006). GAMs were conducted separately for the different seasons cold‐dry and warm‐wet seasons. GAM was computed using the *gam* function in the “mgcv” package with a Gaussian type of family and an “identity” link function. The basis dimensions were reduced to *k* = 5 to avoid over‐parameterization (Peters et al., 2016). For testing the effect of elevation on network indices from cross‐season data, GAM models were constructed using seasonal network interaction indices as the response and elevation as a predictor variable. Since we equally aimed at testing and visualizing the differences of interaction networks in seasons, we included the seasons (cold‐dry and warm‐wet) as factorial variables in respective GAM models as follows and selected a final model using a sequential hierarchical approach:

1. Network ~ Elevation * Season (interactive effect model)
2. Network ~ Elevation + Season (additive effect model)
3. Network ~ Elevation (elevation‐only model)

We started with the interactive effect model, checking for the significance of the interaction term. If the interaction term was not significant, we tested the additive effect model; if the seasonal effect was not significant, we tested the elevation‐only model.

In addition, when patterns associated with elevation gradients were detected, we further tested for each quantitative index (response) the effect of predictor variables most likely to define changes in network structures (*w*NODF, *H2*′, *d*′_(bees)_, *d*′_(plants)_, *Q*, *w*C, *β*_OS_). We examined the impact of climatic (MMT, MMP), flower (*F*_γ_), and bee (*b*_γ,_ log(abun)) parameters on overall network structures. We calculated bee species diversity (*b*_γ_) as the cumulative bee species richness per study plot across the cold‐dry and warm‐wet seasons, respectively (e.g., Dzekashu et al., 2022). To obtain a measure of the diversity of bee‐visited plants, we calculated the species diversity of plants (*F*_γ_) using the same approach as that employed for the bees. Bee abundance, recorded as the total number of visits of bees to plants, was log‐transformed to fit assumptions of normality. We selected final models by applying a multimodal inference (ordinary linear models) framework based on the Akaike Information Criterion (AIC). Multimodal inference frameworks have been shown to objectively account for uncertainties in model selection, parameter estimates, and correlated explanatory variables (Peters et al., 2016, 2019). Furthermore, we used ordinary linear models to predict the effect of drivers on network structures because we expect that network metrics respond in a linear way to environmental predictor variables, e.g., increase with temperature in a monotonic way. Because our sample size was small compared with the number of estimated parameters (*n*/*K* < 40), we used the Akaike Information Criterion (AIC_C_) with second‐order biased correction rather than the original AIC to descend support for individual models (Burnham & Anderson, 2002). We standardized all independent and response variables by *z*‐transformation to allow for direct and comprehensive appraisals of the effect size among regressors. We assembled a full model for each response variable, comprising climate, bee, and plant diversity variables, and calculated AIC_C_ values for these and all nested models. To select the best model, we equated all models presenting a ΔAIC_C_ < 3.

We used Pearson correlation coefficients to check for collinearity among predictor variables (Figure 6). Across the studied elevation gradient, the correlation between *F*_γ_ and *b*_γ_ was higher than *r* > .7 (Dormann et al., 2013) and we note that this can cause glitches in causal inference. We analyzed them together to quantify their comparative support as predictor variables in the multi‐model inference approach but note that the influence of the best‐supported predictor variables has to be carefully interpreted by considering correlated diversity variables.

## 3 RESULTS

We recorded 16,741 interactions between 186 bee (pollinator) morphospecies (hereafter termed “species”) and 314 plant species. Five of the six families of bees known to exist in East Africa were recorded on plants during this study (Tables S4 and S5). Overall, bees in the family Apidae were the most frequent plant visitors with 14,988 interactions (89.5%). The Western honeybee (*Apis mellifera* [*Am*]) constituted the highest number of visits to flowers (13,619 interactions, 81.4%), while other bees in the family Apidae (excluding *Am*) affected 1369 (8.1%) interactions. This was followed by bees from the family Halictidae (1253 interactions, 7.5%), then the families Megachilidae (439 interactions, 2.6%), Colletidae (56 interactions, 0.33%), and Andrenidae (5 interactions, 0.03%).

### 3.1 Seasonal and elevational patterns of network interaction

**FIGURE 2:**
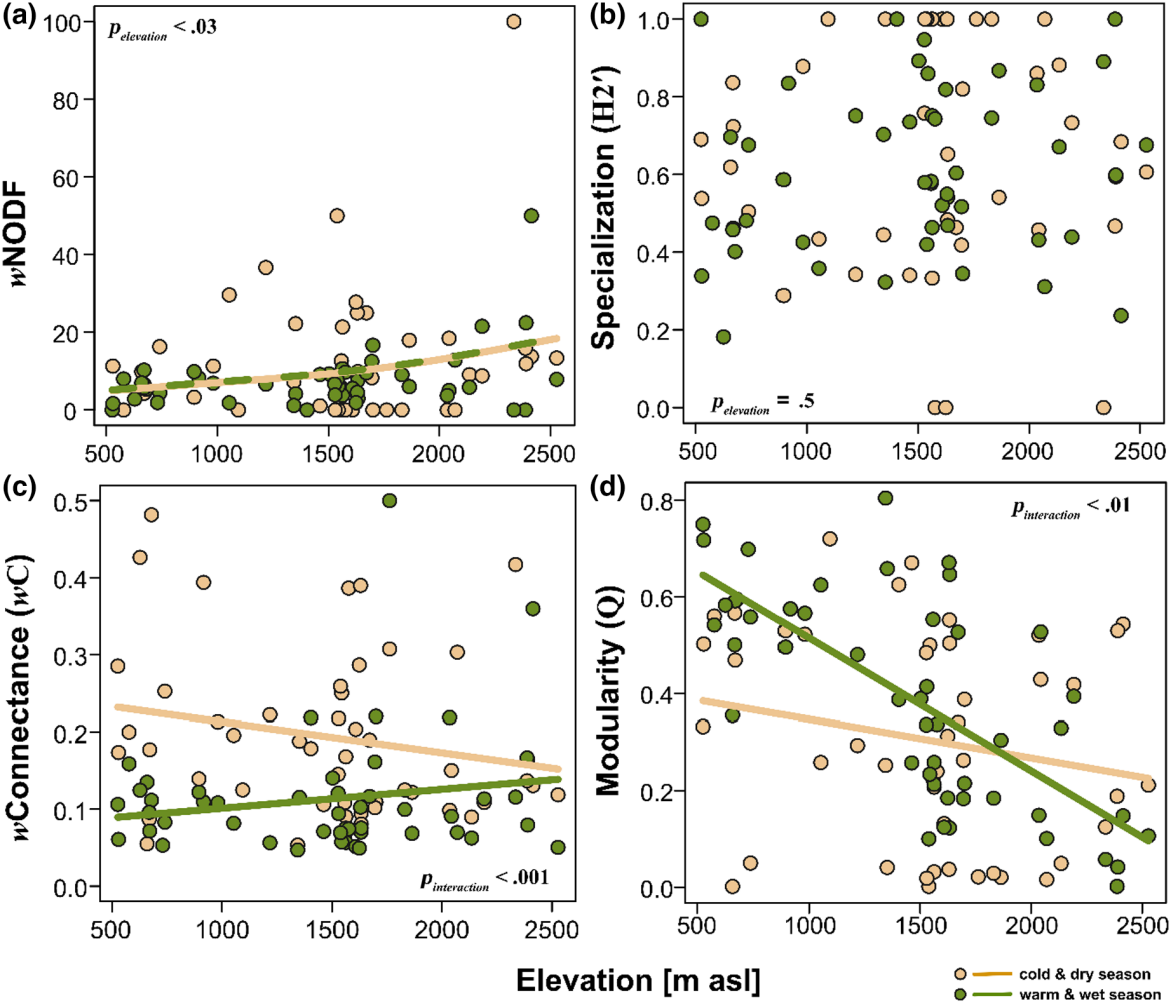
Seasonal and elevational patterns of bee‐plant interaction network indices. (a) Patterns of Weighted Nestedness Overlap and Decreasing Fills (*w*NODF) with elevation. The values range from 0 to 100. High values indicate higher nestedness and lower values the inverse. (b) Patterns of network specialization (ranges from 0 to 1) with elevation, high values indicate increased network specialization. (c) Patterns of weighted network connectance (*w*C) with elevation, high values indicate increased levels of realized interactions. (d) Patterns of network modularity (*Q*) with elevation. Higher values indicate increased interactions among link‐rich clusters (ranges from 0 to 1). All seasonal network trends were analyzed using generalized additive models (Gaussian family, basis dimension *k* = 5). The *p*‐values within boxes indicate the statistical differences for each network index between the two seasons across elevation (i.e., cold‐dry and warm‐wet). Intermittent yellow and green trend lines indicate no significant interactive effects between seasonal networks (i.e., cold‐dry and warm‐wet).

**FIGURE 3:**
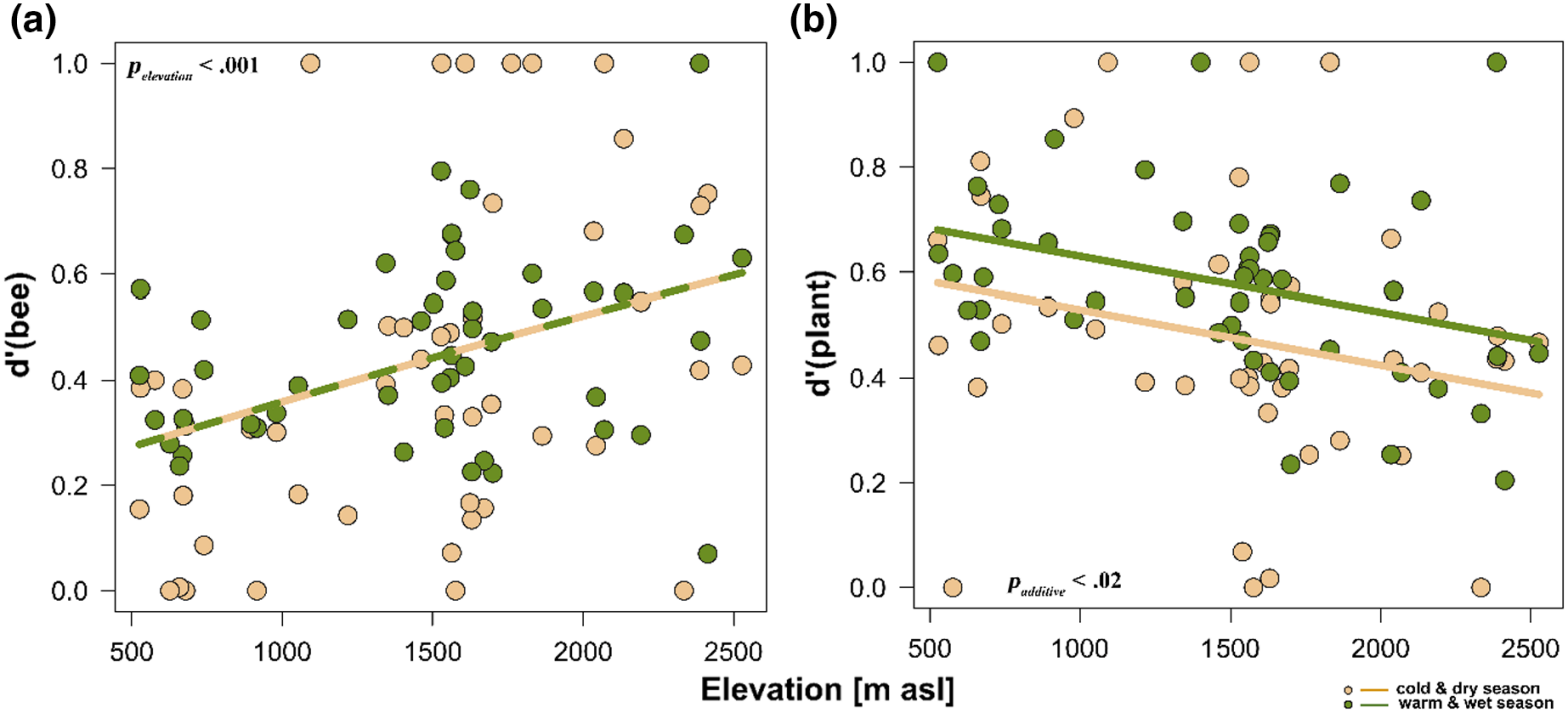
Seasonal and elevational patterns of species‐level specialization of plant‐bee interaction networks across elevation. (a) Seasonal patterns of species specialization of bees with elevation (ranges from 0 to 1). (b) Seasonal patterns of species specialization of plants with elevation (ranges from 0 to 1). High values indicate increased specialization. All seasonal network trends were analyzed using generalized additive models (Gaussian family, basis dimension *k* = 5). The *p*‐values within boxes indicate the statistical differences for each network index between the two seasons across elevation (i.e., cold‐dry and warm‐wet). Intermittent yellow and green trend lines indicate no significant interactive effects between seasonal networks (i.e., cold‐dry and warm‐wet).

**FIGURE 4:**
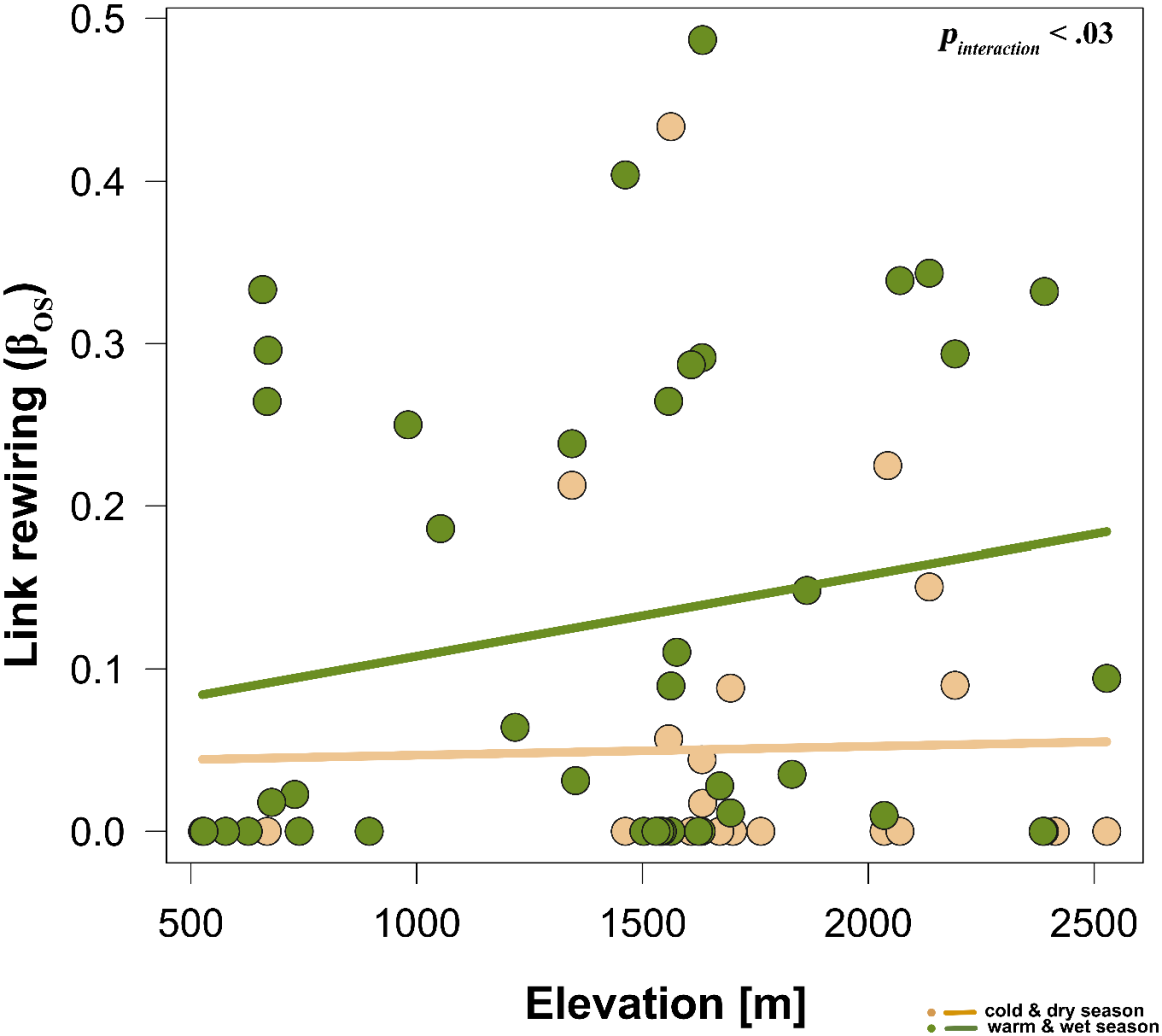
Seasonal and elevational pattern of interaction rewiring across elevation. Higher values indicate increased link rewiring (ranges from 0 to 1). The seasonal network trend was analyzed using generalized additive models (Gaussian family, basis dimension *k* = 5). The *p*‐values within the box indicate the statistical differences for interaction dissimilarity (link rewiring) between the two seasons across elevation (i.e., cold‐dry and warm‐wet).

Our results highlighted the relevance of season for the structure of plant‐bee interaction networks, with stronger patterns observed during the warm‐wet than in the cold‐dry seasons (Figures S3 and S4). We found that, on average, *w*NODF marginally increased with elevation (*n* = 93, Explained deviance (ED) = 7.8%, *F*_elevation_ = 3.14, *p*_elevation_ = .03, Figure 2a) but did not differ across the cold‐dry and warm‐wet seasons. Network specialization (*H2*′) did not change with elevation (*n* = 90, ED = 0.5%, *F*_elevation_ = 0.45, *p*_elevation_ = .5, Figure 2b), and the pattern was the same across the cold‐dry and warm‐wet seasons (Figure 2b). There was a contrasting pattern in network connectance across seasons, such that, there was a high number of realized interactions at lower elevations during the cold‐dry season, while more interactions were realized towards the higher elevations during the warm‐wet season (*n* = 97, ED = 19.1%, *F*_interaction_ = 10.91, *p*_interaction_ = .001, Figure 2c). Network modularity equally differed across seasons such that, there was an increase in interactions among link‐rich clusters at lower elevations in the warm‐wet seasons than in the cold‐dry seasons (*n* = 93, ED = 30.3%, *F*_interaction_ = 5.36, *p*_interaction_ = .01, Figure 2d). The degree of species‐level specialization of bees (*d*'_bees_) increased exponentially across the elevation gradient (*n* = 95, ED = 13.4%, *F*_elevation_ = 12.49, *p*_elevation_ = .001, Figure 3a) with no significant difference between seasons (Figure 3a). On the contrary, the patterns of species‐level specialization of plant species (*d*'_plants_) differed across seasons such that higher plant species‐level specialization was observed during the warm‐wet than in the cold‐dry seasons, with both patterns marginally declining with increases in elevation (*n* = 91, ED = 12.8%, *F*_additive_ = 7.05, *p*_additive_ = .02, Figure 3b). We also revealed that interaction reassembly or link rewiring (*β*_OS_) between networks differed across seasons such that, there was a gain in seasonal reassembled interactions with elevation during the warm‐wet seasons; however, this was followed by a reduced and insignificant trend during the cold‐dry season (*n* = 67, ED = 10.4%, *F*_interaction_ = 3.54, *p*_interaction_ < .03, Figure 4).

The observed trends were largely robust towards the exclusion of plots with low numbers of sampled species interactions (Figure S2). Only for *w*NODF and *d*'_bee_ slight changes in elevational patterns were observed, with no change of *w*NODF in the cold‐dry season and a steeper slope in *d*'_bee_ in the cold‐dry season (Figure S2).

### 3.2 Seasonal and elevational patterns of bees and plants assemblages

**FIGURE 5:**
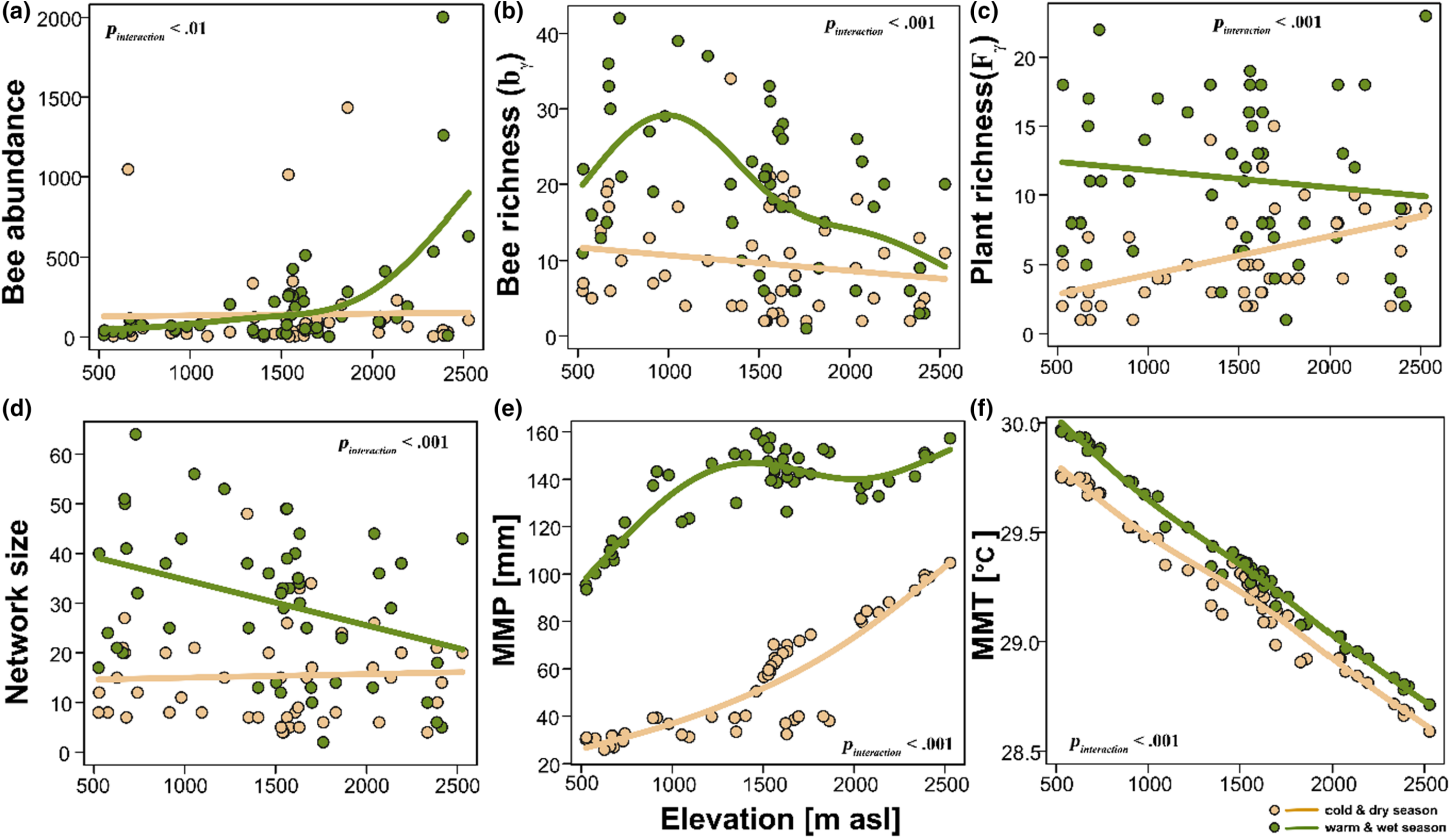
Patterns of bees, plants, and climatic variables used to explain variations in network topologies. (a) Patterns of bee species abundance with elevation. (b) Patterns of bee species richness with elevation. (c) Patterns of plant species richness with elevation. (d) Patterns of interaction network size across elevations. (e) Patterns of mean monthly precipitation (MMP) with elevation. (f) Patterns of mean monthly temperature (MMT) with elevation. All seasonal diversity trends were analyzed using generalized additive models (Gaussian family, basis dimension *k* = 5). The *p*‐values within boxes indicate the statistical differences for each network index between the two seasons across elevation (i.e., cold‐dry and warm‐wet).

Elevation had a strong influence on network assembly patterns. We recorded individual plots between 1 and 23 plant species visited by 1 and 42 bee species (Table S3). We found that bee abundance differed across seasons such that bee abundance increases exponentially with increasing elevation from ~1700 m asl during the warm‐wet but not during the cold‐dry seasons (*n* = 97, Explained deviance (ED) = 24.9%, *F*_interaction_ = 4.46 *p*_interaction_ < .01, Figure 5a). We equally found that bee species richness decreases with increased elevation and exhibited a hump‐shape pattern with elevation during the warm‐wet season with peaks at ~1100 m asl. Moreover, bee species richness differed across seasons such that bee species richness was higher during the warm‐wet than in the cold‐dry seasons (*n* = 97, ED = 39.4%, *F*_interaction_ = 8.99, *p*_interaction_ < .001, Figure 5b). Patterns of plant species richness were contrasting (i.e., increasing and decreasing) with elevation and across seasons, such that a conspicuous trend of decreasing plant species richness with elevation was resolved during the warm‐wet seasons while the opposite trend occurred during the cold‐dry seasons (*n* = 97, ED =30.4%, *F*_interaction_ = 19.9, *p*_interaction_ < .001, Figure 5c). Network size equally dif fered across seasons and elevation, such that, more networks were realized during the warm‐wet seasons at lower elevations, while less networks were realized in the cold‐dry seasons with no noticeable differences along the elevation gradient (*n* = 97, ED = 33.1%, *F*_interaction_ = 21.02, *p*_interaction_ < .001, Figure 5d). Moreover, while we found mean monthly precipitation amount to exponentially increase with increasing elevation in both cold‐dry and warm‐wet seasons (*n* = 100, Explained deviance (ED) = 96.2%, *F*_interaction_ = 419.8, *p*_interaction_ = .001, Figure 5e), we, however, observed a monotonically decrease in mean monthly temperature along the elevation gradient in both cold‐dry and warm‐wet seasons (*n* = 100, ED = 98%, *F*_interaction_ = 60.4, *p*_interaction_ = .001, Figure 5f), respectively.

### 3.3 Drivers of seasonal plant‐bee pollinator network patterns

**FIGURE 6:**
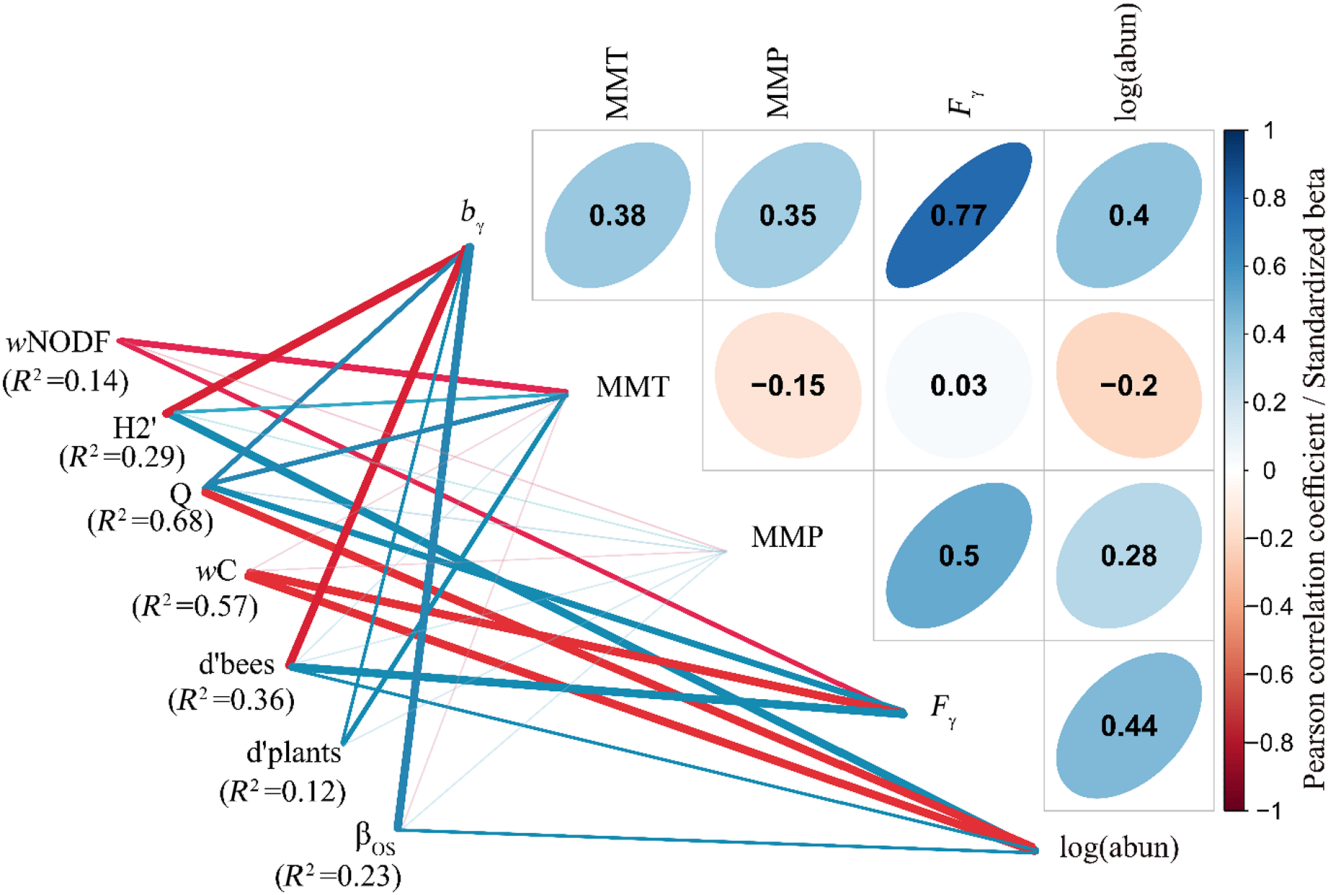
Summary of best‐fit models. This depicts significant predictors of plant‐bee interaction networks along the elevation gradients for the different network indices measured. The boldness of individual links represents the relative strength of an association, while the colors blue and red signify positive and negative interacting effects. The relative amount of explained variance or coefficient of variation (*R* ^2^) is specified for each response variable. The correlation matrix (correlogram) on the right highlights the direction and strength in the relationship between explanatory variables, which are bee species diversity (*b*_γ_), mean monthly temperature (MMT), mean monthly precipitation (MMP), flower species diversity (*F*_γ_), and recorded abundance of all bees on each study plot (log(abun)).

Bee species richness, flower resource richness, and climate were identified as main predictors of the observed network structures across the elevational gradients. The Weighted Nestedness Overlap and Decreasing Fills (*w*NODF) was negatively influenced by increases in temperature (MMT) and flower diversity (*F*_γ_), and slightly negatively by precipitation (MMP) (Figure 6, Table S6). However, the support for this was weak (*R* ^2^ = 14%), indicating that the network structure could be shaped by neutral processes, resulting in more randomly nested networks at higher elevations. Network specialization (*H2*′) was significantly and negatively influenced by bee species diversity (*b*_γ_) but strongly and positively by bee abundance and marginally by temperature (MMT). However, network modularity was higher in dry areas and was positively correlated with flower, bee species richness, temperature (MMT), and slightly by precipitation (MMP) but strongly and negatively with bee species abundance (Figure 6, Table S6). Our results equally revealed that network connectance was negatively and significantly related to the abundance of bee species and richness of flower species (*F*_γ_) (Figure 6). Bee species specialization was strongly and significantly predicted by floral species diversity (*F*_γ_) and bee species diversity (*b*_γ_), with bee species specialization increasing under favorable conditions with flower species richness and bee species abundance but decreasing in areas with low bee species diversity (Figure 6, Table S6). On the other hand, plant species specialization strongly and significantly increased with bee species diversity (*b*_γ_), temperature (MMT), and slightly with precipitation amounts (MMP) but decreases in areas of low flower diversity (*F*_γ_). Furthermore, the average trends in seasonal reassembled interactions (*β*_OS_) were positively and strongly influenced by bee species diversity and abundance (Figure 6, Table S6).

## 4 DISCUSSION

Our study on plant‐bee pollinator interaction networks along two mountain slopes in the Eastern Afromontane Biodiversity Hotspots (EABH) revealed significant changes in plant‐pollinator interaction networks with both elevation and season. Using our quantitative data set, we unraveled how climatic variables, diversity, and changes in species communities influenced network structures.

### 4.1 Elevational trends in plant‐bee interaction networks

At lower elevations, networks were more nested, but the trend became random at higher elevations such that specialized species tend to interact with subsets of highly generalized partners. These results mirror that of studies carried out on Mt. Kilimanjaro and Mt. Cameroon (Classen et al., 2020; Mertens et al., 2021). It is equally in line with other studies investigating constancy or stability in ecosystems (e.g., Albrecht et al., 2010). Here, due to reduced preferred feeding resources, bees with specialized feeding requirements shift their foraging to lower elevations with enough varieties of feeding resources, thus leaving the set of generalist feeders unaltered at high elevations (Tylianakis et al., 2010). However, nestedness is considered as a typical interaction property for more stable ecological networks (Albrecht et al., 2010; Bascompte & Jordano, 2007), which predominantly would occur at higher than at lower elevations.

Whereas species‐level specialization of bee species increased with elevation, a decreasing pattern emerged for plant species specialization. This general pattern, however, differs from reports of plant‐pollinator networks along tropical elevations (Classen et al., 2020), where a linearly and decreasing pattern in network, bee, and plant species‐level specialization with elevation was observed. One possible explanation of this finding is that, on average, bee species specialization was much higher in our studied networks than in the pollination networks of Classen et al. (2020), because beekeeping activities were carried out by local indigenes in the forested highlands of our study area (See Figure S1). Higher elevations are normally characterized by reductions in species richness (Peters et al., 2016), hence, we would expect a decrease in interspecific competition for resource usage at higher elevations. However, bees, especially honeybees (*Apis mellifera*), are known to show a high degree of floral constancy (Goulson, 1999; Ivey et al., 2003), and as such would continue foraging on specific flowering plants with abundant resource rewards that can equally offset the energy cost required in obtaining similar rewards from different environments (Harrison & Winfree, 2015). Therefore, an increase in flower constancy as a result of interspecific competition, and the availability of abundant and highly nutritious flower resources at higher elevations may influence bee foraging decisions, thereby, leading to high species‐level specialization (Lawlor & Smith, 1976; Suni et al., 2022) of bees observed in the highlands.

Our results equally revealed a contrasting pattern in realized interactions (connectance) with elevation. This trend was highest at the lower elevations in the cold‐dry and highest in the highlands during the warm‐wet seasons. Previous studies in the Cape Floristic Region of South Africa have shown network connectance to be high at higher elevations (Adedoja et al., 2018). Increased network connectance is known to increase the core links of generalists in a pollination network (Olesen et al., 2007) and improve robustness in ecological communities (Dunne et al., 2002). A plausible explanation here could be that plant dependence on pollinators for pollination services would result in them being more sensitive to pollinator loss at higher elevations. Also, bee species are less tolerant to the fluctuation of more sensitive generalist plants (Kaiser‐Bunbury et al., 2010). Hence, the few available plant species along this elevation gradient would be visited by a host of generalists' bee species leading to an increase in realized interactions.

Modularity, on the other hand, linearly and significantly decreases with elevation. However, this contradicts results from previous studies of plant‐pollinator interactions from the Andes (Ramos‐Jiliberto et al., 2010) but mirrors those from the Canary Islands (Lara‐Romero et al., 2019) and Mt. Cameroon (Mertens et al., 2021). High modularity is known to occur when modules appear isolated from the rest of the network (Beckett, 2016). This binds diverse subgroups within individual networks and increases the stability of pollinator systems by buffering the effects of perturbations across link‐rich clusters (Tylianakis et al., 2010). If such effects remain unchecked, they are capable of causing longstanding effects on network topologies and organization (Olesen et al., 2007). As such, we can therefore attribute the exponential decrease in modularity to the decrease in species richness along the elevation gradient, indicative of the competitive‐mediated effects of available floral resources for bees (Spiesman & Gratton, 2016).

Link rewiring on the other hand, linearly and significantly increases with elevation. Rewiring in less diverse communities, such as at higher elevations, made up of fewer interacting species can increase functional redundancy in the community (Kühsel & Blüthgen, 2015) by limiting species loss through host switching when the available plant species are visited by several bees occurring in these communities. In our study, the exponential increase in link rewiring can be attributed to favorable climatic conditions. Here, climate‐mediated phenological differences and adaptive foraging would shape rewiring patterns of pollination network communities (Kaiser‐Bunbury et al., 2010; Vázquez, 2005). As such, the formation of many new interactions as a result of seasonal changes coupled with the pliability of generalist bees and plants across the elevation gradient would provide some stability and robustness in the face of extinction cascades (Burkle et al., 2013).

### 4.2 Seasonal trends in plant‐bee interaction networks

The plant‐bee pollination networks were distinct across seasons (Figure 5a–c). Seasonality is known to shape the period of species occurrence and their interaction patterns. More so, a detailed seasonal turnover of bees and bee‐visited plant species has been reported for this region (see Dzekashu et al., 2022). Here, we observed an increase in nestedness with elevation, though it did not differ across seasons, following similar patterns of complete network interactions described above.

Our results further revealed no considerable elevational and seasonal change for network specialization. This corroborates the findings of other studies from a global meta‐analysis (Schwarz et al., 2020) and plant‐hoverflies interaction in temperate regions (de Manincor et al., 2020) but differs from a plant‐Lepidoptera interaction study in the tropics (Mertens et al., 2021). This can be because of lengthier phenophases of some species, which will lead to the accumulation of additional links over time hence reducing the specialization pattern (Schwarz et al., 2020). Even though, seasonal segregation and high species turnover would have resulted in limited interacting species, these networks are more specialized across the elevation gradient (Schwarz et al., 2020).

There were strong contrasting patterns in bee and plant species specialization, leading to high bee species specialization at high elevations across all seasons and high plant species specialization at low elevations across all seasons with a noticeably increased trend during the warm‐wet than in the cold‐dry seasons. This can be explained by the fact that this region harbors very extreme vegetation and climatic conditions across the different seasons. During the cold‐dry season, when temperatures are extremely high, there is an overall dry vegetation with very few or no available flowering plants and food resources for bees at lower elevations, thus leading to a massive shift in bee foraging range to ~350 m asl upslope (Dzekashu et al., 2022). However, more plants would turn to bloom at lower elevations when conditions become favorable, during the warm‐wet season (Dzekashu et al., 2022).

We equally noticed a strong contrasting pattern in network connectance, with high connectance at low elevations during the cold‐dry season and high connectance at high elevations during the warm‐wet seasons. A previous study (Ramos‐Jiliberto et al., 2010) found connectance to increase with altitude. We can argue this to be a result of reduced interdependence at higher elevations due to the increase in generality, hence the increasing connectance during the warm‐wet season (Classen et al., 2020). Moreover, the high seasonal turnover of species along these elevation gradients (Dzekashu et al., 2022) might prevent the aggregation of a more compact structure, hence the inconsistencies in observed connectance across seasons.

Our results equally revealed significantly different patterns in network modularity across seasons, such that the trends were higher in the warm‐wet than in the cold‐dry seasons. This pattern illustrates that seasonal shifts in species diversity did not result in a decline in network modularity. As such, robustness mechanisms against extinction cascades can be considered well maintained across the different seasons as observed along the elevation gradients of this region.

Patterns of link rewiring changed across seasons. As such, more links were established during the warm‐wet than in the cold‐dry seasons. This can be attributed to the seasonal turnover in species assemblages, which would lead to increased link rewiring with more subsets of species eventually contributing to higher generalization and connectance (Schwarz et al., 2020).

### 4.3 Drivers of plant‐bee network patterns

Network structures were shaped by a plethora of factors among which are plant diversity, bee diversity, and climate. These factors influenced temporal fluctuations in bees and flowering plants, leading to a modification in interaction efficiency between co‐occurring species.

Nestedness increased across the elevation since specialist species were more likely to interact with generalist species at higher elevations, a pattern negatively influenced by mean monthly temperature and flower species diversity. We argue that favorable climatic conditions would enable temporal fluctuations in bee and plant species, thereby leading to increased interaction efficiency. Moreover, climate can also have an effect on nestedness patterns via network rewiring, whereby, suitable climatic conditions would lead to plant proliferation, hence the formation of more realized links between subsets of similar species (Schwarz et al., 2020).

The mechanisms contributing to network specialization were likewise sturdy, such that an increase in bee species diversity led to a reduction in network and degree of bee specialization (*H2*, *d*′_(bees)_). Plant diversity nonetheless positively influenced specialization in bee species. As such, plants in species‐rich assemblages with high dissemination rates can reduce pollen loss with the help of specialized pollinators. However, high competition in more diverse low elevations between interacting partners can enhance segregation among co‐occurring species leading to the establishment of smaller niches thereby increasing specialization (Hoiss et al., 2015).

We noticed that the observed modular structure was positively influenced by temperature, bee, and floral diversity but negatively influenced by bee species abundance. This confirmed our earlier findings that higher elevations in this region are characterized by low plant diversity (Dzekashu et al., 2022). Bee species diversification occurs faster than plant diversity across elevations, thereby limiting competition between bee species at higher elevations. Thus, bees with high resource and energy demands turn to expand their feeding spectrum to other more favorable plant species at lower elevations (Hoiss et al., 2015).

Furthermore, low temperatures at higher elevations constraint resource acquisition for bees with high metabolic demand for energy intake and population growth (Classen et al., 2015; Savage et al., 2004). As a result, the diversification of ectotherms is high at lower and warm elevations (Peters et al., 2019).

Our findings showed that across seasons, more links were realized among subsets of interacting species leading to increased connectance. However, increased interdependence among co‐occurring species would strongly constrained network connectance across the elevation gradient (Thébault & Fontaine, 2010). Moreover, patterns in network link rewiring were positively influenced by bee species diversity and abundance. Here, and across seasons, sites rich in rainfall and bee species diversity promote the proliferation of more plant species either indirectly via pollen deposition and or pollination success due to increased generalization hence the high network rewiring at higher elevations.

## 5 CONCLUSION

We found significant changes for all network structures except network specialization with elevation. Partitioning networks into seasonal components revealed a marked shift in network architecture across seasons. These structural changes across season and elevation can partly be explained by link rewiring, whereby more links are realized among subsets of interacting species leading to the observed trends across seasons and elevation. The observed trends equally point to the influence of environmental filters (changing climate) as factors shaping these assemblages. Tropical elevations are characterized by an interplay of fluctuating climate and floral resource availability, which can lead to some more synchronized interactions because bees are fully dependent on available floral resources. Therefore, reduced seasonal variation in temperature and precipitation could lead to phenological mismatches between interaction partners along elevation gradients of the EABH. We thus emphasize the urgent need for conservation and restoration efforts aimed at reducing climate change effects and harnessing the ability of mutualistic organisms to restore broken links in order to improve ecosystem resilience and functioning along the slopes of the Eastern Afromontane Biodiversity Hotspots.

## AUTHOR CONTRIBUTIONS

**Fairo Foryuy Dzekashu:** Conceptualization (equal); data curation (lead); formal analysis (lead); investigation (lead); methodology (lead); software (lead); validation (lead); visualization (lead); writing – original draft (lead); writing – review and editing (lead). **Christian Pirk:** Conceptualization (equal); investigation (equal); supervision (lead); validation (equal); writing – review and editing (equal). **Abdullahi Ahmed Yusuf:** Conceptualization (equal); investigation (equal); methodology (equal); supervision (equal); validation (equal); writing – review and editing (equal). **Alice Classen:** Investigation (equal); validation (equal); visualization (equal); writing – review and editing (equal). **Nkoba Kiatoko:** Supervision (equal); writing – review and editing (equal). **Ingolf Steffan‐Dewenter:** Supervision (equal); validation (equal); visualization (equal); writing – review and editing (equal). **Marcell Karl Peters:** Formal analysis (equal); methodology (equal); supervision (equal); validation (equal); visualization (equal); writing – review and editing (equal). **H. Michael G. Lattorff:** Conceptualization (equal); funding acquisition (lead); investigation (equal); methodology (equal); project administration (lead); resources (lead); supervision (equal); writing – review and editing (equal).

### OPEN RESEARCH BADGES

This article has earned an Open Data badge for making publicly available the digitally‐shareable data necessary to reproduce the reported results. The data is available at https://doi.org/10.25403/UPresearchdata/19763545 (Dzekashu et al., 2023).

## Supporting information


Data S1.

## Data Availability

All data supporting this study are available from Figshare: https://doi.org/10.25403/UPresearchdata/19763545 (Dzekashu et al., 2023).
