# Supplementary material for: Seasonal and elevational changes of plant‐pollinator interaction networks in East African mountains
Source: Ecol Evol. 2023 May 11;13(5):e10060. doi: 10.1002/ece3.10060 (PMC10175727; doi:10.1002/ece3.10060)
Supplement: Supplementary file 1 — Data S1. [file ECE3-13-e10060-s001.docx]

**SUPPORTING INFORMATION**

Seasonal and elevational changes of plant-pollinator interaction networks in East African mountains

**OPEN DATA STATEMENT**

All data supporting this study are available on Figshare at; <https://figshare.com/s/dc50c1a08540f41fbb96>

A DOI will be generated and made available upon acceptance of this manuscript.

**Table S1**. Monthly timeseries climatic variables obtained from the CHELSA database (Modified from Karger et al., 2017 and Brun et al., 2022)

| **Shortname** | **Shortname CHELSA** | **Longname** | **Unit** | **Explanation** |
| --- | --- | --- | --- | --- |
| MMT | tas_03, tas_04, tas_06, tas_09, tas_10, tas_11, tas_12 | mean daily air temperature for each month | °C/10 | Daily mean air temperature at 2 metres from hourly ERA5 data for each month |
|  |  |  |  |  |
| MMP | pr_03, pr_04, pr_06, pr_09, pr_10, pr_11, pr_12 | monthly precipitation amount | kg m^-2^ month^-1^ /100 | Precipitation amount for each month; "Amount" means mass per unit area. "Precipitation" in the Earth's atmosphere means precipitation of water in all phases |

**Table S2:** Geographical locations of study sites (Murang’a and Taita Taveta counties, Kenya), including plot codes, elevation, and coordinates (WGS 84).

| **Site** | **Plot code** | **Elevation** | **Latitude** | **Longitude** |
| --- | --- | --- | --- | --- |
| Murang'a | M1U1 | 2414 | -0.74884 | 36.7914 |
| Murang'a | M1U2 | 2387 | -0.73577 | 36.799058 |
| Murang'a | M1U3 | 2335 | -0.76688 | 36.78757 |
| Murang'a | M1U4 | 2528 | -0.70896 | 36.787407 |
| Murang'a | M1U5 | 2390 | -0.71752 | 36.808793 |
| Murang'a | M2U1 | 2135 | -0.80727 | 36.839151 |
| Murang'a | M2U2 | 2043 | -0.8277 | 36.867321 |
| Murang'a | M2U3 | 2035 | -0.81184 | 36.888618 |
| Murang'a | M2U4 | 2192 | -0.77118 | 36.84008 |
| Murang'a | M2U5 | 2070 | -0.78225 | 36.87113 |
| Murang'a | M3U1 | 1762 | -0.86113 | 36.974227 |
| Murang'a | M3U2 | 1700 | -0.88759 | 36.984516 |
| Murang'a | M3U3 | 1558 | -0.89655 | 37.010799 |
| Murang'a | M3U4 | 1632 | -0.90315 | 36.985293 |
| Murang'a | M3U5 | 1563 | -0.95437 | 37.018954 |
| Murang'a | M4U1 | 1633 | -0.92742 | 37.012291 |
| Murang'a | M4U2 | 1608 | -0.9333 | 37.038271 |
| Murang'a | M4U3 | 1563 | -0.96892 | 37.038756 |
| Murang'a | M4U4 | 1576 | -0.97247 | 37.003061 |
| Murang'a | M4U5 | 1544 | -0.98592 | 37.024367 |
| Murang'a | M5U1 | 1539 | -0.96254 | 37.119629 |
| Murang'a | M5U2 | 1503 | -0.98144 | 37.129597 |
| Murang'a | M5U3 | 1462 | -0.99932 | 37.184546 |
| Murang'a | M5U4 | 1528 | -0.99307 | 37.075808 |
| Murang'a | M5U5 | 1530 | -0.96727 | 37.096123 |
| Taita Taveta | T1U1 | 1864 | -3.35813 | 38.338466 |
| Taita Taveta | T1U2 | 1831 | -3.39831 | 38.337664 |
| Taita Taveta | T1U3 | 1671 | -3.48964 | 38.34585 |
| Taita Taveta | T1U4 | 1695 | -3.43159 | 38.338236 |
| Taita Taveta | T1U5 | 1630 | -3.33344 | 38.446883 |
| Taita Taveta | T2U1 | 1218 | -3.45556 | 38.353352 |
| Taita Taveta | T2U2 | 1352 | -3.35776 | 38.430439 |
| Taita Taveta | T2U3 | 1624 | -3.34152 | 38.321148 |
| Taita Taveta | T2U4 | 1404 | -3.42846 | 38.357807 |
| Taita Taveta | T2U5 | 1344 | -3.39909 | 38.364769 |
| Taita Taveta | T3U1 | 1094 | -3.31216 | 38.433244 |
| Taita Taveta | T3U2 | 1053 | -3.39758 | 38.429537 |
| Taita Taveta | T3U3 | 981 | -3.39698 | 38.389197 |
| Taita Taveta | T3U4 | 916 | -3.46425 | 38.376484 |
| Taita Taveta | T3U5 | 895 | -3.50153 | 38.391315 |
| Taita Taveta | T4U1 | 740 | -3.4208 | 38.423796 |
| Taita Taveta | T4U2 | 730 | -3.31954 | 38.484921 |
| Taita Taveta | T4U3 | 679 | -3.27704 | 38.493254 |
| Taita Taveta | T4U4 | 671 | -3.4358 | 38.455915 |
| Taita Taveta | T4U5 | 669 | -3.44498 | 38.501685 |
| Taita Taveta | T5U1 | 659 | -3.27078 | 38.453469 |
| Taita Taveta | T5U2 | 627 | -3.2511 | 38.478018 |
| Taita Taveta | T5U3 | 577 | -3.45436 | 38.638178 |
| Taita Taveta | T5U4 | 526 | -3.48225 | 38.671232 |
| Taita Taveta | T5U5 | 529 | -3.51132 | 38.698079 |

**Table S3:** Bee species abundance, bee species richness, plant species richness and network size (Bee species richness + plant species richness) per study plot during the cold-dry and warm-wet seasons.

| **Plots** | **Season** | **Bee abundance** | **Bee richness** | **Plant richness** | **Network size** |
| --- | --- | --- | --- | --- | --- |
| M1U1 | cold-dry | 30 | 5 | 9 | 14 |
| M1U2 | cold-dry | 43 | 13 | 8 | 21 |
| M1U3 | cold-dry | 4 | 2 | 2 | 4 |
| M1U4 | cold-dry | 106 | 11 | 9 | 20 |
| M1U5 | cold-dry | 10 | 4 | 6 | 10 |
| M2U1 | cold-dry | 230 | 5 | 10 | 15 |
| M2U2 | cold-dry | 127 | 18 | 8 | 26 |
| M2U3 | cold-dry | 28 | 9 | 8 | 17 |
| M2U4 | cold-dry | 65 | 11 | 9 | 20 |
| M2U5 | cold-dry | 121 | 2 | 4 | 6 |
| M3U1 | cold-dry | 91 | 2 | 4 | 6 |
| M3U2 | cold-dry | 58 | 8 | 9 | 17 |
| M3U3 | cold-dry | 253 | 17 | 16 | 33 |
| M3U4 | cold-dry | 55 | 21 | 12 | 33 |
| M3U5 | cold-dry | 350 | 21 | 5 | 26 |
| M4U1 | cold-dry | 84 | 18 | 12 | 30 |
| M4U2 | cold-dry | 43 | 3 | 5 | 8 |
| M4U3 | cold-dry | 34 | 5 | 2 | 7 |
| M4U4 | cold-dry | 5 | 3 | 2 | 5 |
| M4U5 | cold-dry | 2 | 2 | 2 | 4 |
| M5U1 | cold-dry | 1013 | 2 | 2 | 4 |
| M5U2 | cold-dry | NA | NA | NA | NA |
| M5U3 | cold-dry | 19 | 12 | 8 | 20 |
| M5U4 | cold-dry | 24 | 10 | 5 | 15 |
| M5U5 | cold-dry | 105 | 2 | 3 | 5 |
| T1U1 | cold-dry | 1434 | 14 | 10 | 24 |
| T1U2 | cold-dry | 203 | 4 | 4 | 8 |
| T1U3 | cold-dry | 54 | 11 | 4 | 15 |
| T1U4 | cold-dry | 113 | 19 | 15 | 34 |
| T1U5 | cold-dry | 42 | 2 | 3 | 5 |
| T2U1 | cold-dry | 31 | 10 | 5 | 15 |
| T2U2 | cold-dry | 123 | 4 | 3 | 7 |
| T2U3 | cold-dry | 8 | 6 | 3 | 9 |
| T2U4 | cold-dry | 4 | 4 | 3 | 7 |
| T2U5 | cold-dry | 337 | 34 | 14 | 48 |
| T3U1 | cold-dry | 5 | 4 | 4 | 8 |
| T3U2 | cold-dry | 78 | 17 | 4 | 21 |
| T3U3 | cold-dry | 20 | 8 | 3 | 11 |
| T3U4 | cold-dry | 36 | 7 | 1 | 8 |
| T3U5 | cold-dry | 25 | 13 | 7 | 20 |
| T4U1 | cold-dry | 55 | 10 | 2 | 12 |
| T4U2 | cold-dry | NA | NA | NA | NA |
| T4U3 | cold-dry | 7 | 6 | 1 | 7 |
| T4U4 | cold-dry | 37 | 17 | 3 | 20 |
| T4U5 | cold-dry | 110 | 20 | 7 | 27 |
| T5U1 | cold-dry | 1046 | 19 | 2 | 21 |
| T5U2 | cold-dry | 25 | 14 | 1 | 15 |
| T5U3 | cold-dry | 5 | 5 | 3 | 8 |
| T5U4 | cold-dry | 17 | 6 | 2 | 8 |
| T5U5 | cold-dry | 15 | 7 | 5 | 12 |
| M1U1 | Warm-wet | 9 | 3 | 2 | 5 |
| M1U2 | Warm-wet | 2002 | 3 | 3 | 6 |
| M1U3 | Warm-wet | 536 | 6 | 4 | 10 |
| M1U4 | Warm-wet | 633 | 20 | 23 | 43 |
| M1U5 | Warm-wet | 1260 | 9 | 9 | 18 |
| M2U1 | Warm-wet | 119 | 17 | 12 | 29 |
| M2U2 | Warm-wet | 92 | 26 | 18 | 44 |
| M2U3 | Warm-wet | 97 | 6 | 7 | 13 |
| M2U4 | Warm-wet | 191 | 20 | 18 | 38 |
| M2U5 | Warm-wet | 412 | 23 | 13 | 36 |
| M3U1 | Warm-wet | 2 | 1 | 1 | 2 |
| M3U2 | Warm-wet | 27 | 6 | 4 | 10 |
| M3U3 | Warm-wet | 142 | 33 | 16 | 49 |
| M3U4 | Warm-wet | 513 | 28 | 16 | 44 |
| M3U5 | Warm-wet | 427 | 31 | 18 | 49 |
| M4U1 | Warm-wet | 39 | 17 | 13 | 30 |
| M4U2 | Warm-wet | 280 | 27 | 13 | 40 |
| M4U3 | Warm-wet | 258 | 20 | 19 | 39 |
| M4U4 | Warm-wet | 187 | 18 | 15 | 33 |
| M4U5 | Warm-wet | 270 | 22 | 7 | 29 |
| M5U1 | Warm-wet | 257 | 21 | 12 | 33 |
| M5U2 | Warm-wet | 23 | 8 | 6 | 14 |
| M5U3 | Warm-wet | 221 | 23 | 13 | 36 |
| M5U4 | Warm-wet | 21 | 6 | 6 | 12 |
| M5U5 | Warm-wet | 76 | 21 | 11 | 32 |
| T1U1 | Warm-wet | 283 | 15 | 8 | 23 |
| T1U2 | Warm-wet | 128 | 9 | 5 | 14 |
| T1U3 | Warm-wet | 53 | 17 | 8 | 25 |
| T1U4 | Warm-wet | 58 | 6 | 7 | 13 |
| T1U5 | Warm-wet | 51 | 26 | 8 | 34 |
| T2U1 | Warm-wet | 205 | 37 | 16 | 53 |
| T2U2 | Warm-wet | 26 | 15 | 10 | 25 |
| T2U3 | Warm-wet | 224 | 17 | 18 | 35 |
| T2U4 | Warm-wet | 17 | 10 | 3 | 13 |
| T2U5 | Warm-wet | 45 | 20 | 18 | 38 |
| T3U1 | Warm-wet NA | | NA | NA | NA |
| T3U2 | Warm-wet | 74 | 39 | 17 | 56 |
| T3U3 | Warm-wet | 64 | 29 | 14 | 43 |
| T3U4 | Warm-wet | 43 | 19 | 6 | 25 |
| T3U5 | Warm-wet | 70 | 27 | 11 | 38 |
| T4U1 | Warm-wet | 71 | 21 | 11 | 32 |
| T4U2 | Warm-wet | 78 | 42 | 22 | 64 |
| T4U3 | Warm-wet | 59 | 30 | 11 | 41 |
| T4U4 | Warm-wet | 95 | 33 | 17 | 50 |
| T4U5 | Warm-wet | 116 | 36 | 15 | 51 |
| T5U1 | Warm-wet | 45 | 15 | 5 | 20 |
| T5U2 | Warm-wet | 22 | 13 | 8 | 21 |
| T5U3 | Warm-wet | 33 | 16 | 8 | 24 |
| T5U4 | Warm-wet | 12 | 11 | 6 | 17 |
| T5U5 | Warm-wet | 41 | 22 | 18 | 40 |

**Table S4:** Plant species list

| **Family** | **Species** |
| --- | --- |
| Malvaceae | *Abutilon fruticosum* |
| Malvaceae | *Abutilon guineense* |
| Malvaceae | *Abutilon longicuspe* |
| Malvaceae | *Abutilon mauritianum* |
| Malvaceae | *Abutilon sp.1* |
| Fabaceae | *Acacia bussei* |
| Fabaceae | *Acacia hockii* |
| Fabaceae | *Acacia senegalensis* |
| Fabaceae | *Acacia sp. 1* |
| Asteraceae | *Acanthospermum glabratum* |
| Asteraceae | *Acanthospermum hispidum* |
| Amaranthaceae | *Achyranthes aspera* |
| Amaranthaceae | *Achyranthes sp. 1* |
| Lamiaceae | *Achyrospermum schimperi* |
| Asteraceae | *Acmella caulirhiza* |
| Lamiaceae | *Aeollanthus repens* |
| Fabaceae | *Aeschynomene schimperi* |
| Ericaceae | *Agauria salicifolia* |
| Asteraceae | *Ageratum conyzoides* |
| Lamiaceae | *Ajuga integrifolia* |
| Asphodelaceae | *Aloe myriacantha* |
| Asphodelaceae | *Aloe secundiflora* |
| Amaranthaceae | *Alternanthera sessilis* |
| Amaranthaceae | *Amaranthus dubius* |
| Commelinaceae | *Aneilemia aequinoctiale* |
| Fabaceae | *Argyrolobium fischeri* |
| Poaceae | *Aristida adscensionis* |
| Asteraceae | *Aspilia mossambicensis* |
| Acanthaceae | *Asystasia mysorensis* |
| Acanthaceae | *Barleria taitensis* |
| Asteraceae | *Bidens pilosa* |
| Asteraceae | *Bidens sp.1* |
| Acanthaceae | *Blepharis edulis* |
| Poaceae | *Bothriochloa insculpta* |
| Poaceae | *Brachiaria sp.1* |
| Loganiaceae | *Buddleja polystachya* |
| Fabaceae | *Caesalpinia decapetala* |
| Fabaceae | *Calliandra houstoniana* |
| Myrtaceae | *Callistemon viminalis* |
| Apocynaceae | *Calotropis procera* |
| Resedaceae | *Caylusea abyssinica* |
| Fabaceae | *Chamaecrista mimosoides* |
| Fabaceae | *Chamaecrista sp.1* |
| Vitaceae | *Cissus aralioides* |
| Verbenaceae | *Clerodendrum eriophyllum* |
| Lamiaceae | *Clinopodium abyssinicum* |
| Commelinaceae | *Commelina benghalensis* |
| Commelinaceae | *Commelina sp.1* |
| Convolvulaceae | *Convolvulus kilimandschari* |
| Asteraceae | *Conyza bonariensis* |
| Asteraceae | *Conyza newii* |
| Asteraceae | *Conyza schimperi* |
| Asteraceae | *Conyza steudelii* |
| Boraginaceae | *Cordia africana* |
| Brassicaceae | *Crambe cordifolia* |
| Asteraceae | *Crassocephalum montuosum* |
| Asteraceae | *Crassocephalum picridifolium* |
| Asteraceae | *Crassocephalum vitellinum* |
| Fabaceae | *Crotalaria axillaris* |
| Fabaceae | *Crotalaria barkae* |
| Fabaceae | *Crotalaria ukambensis* |
| Euphorbiaceae | *Croton bonplandianus* |
| Cucurbitaceae | *Cucumis dipsaceus* |
| Amaranthaceae | *Cyathula cylindrica* |
| Amaranthaceae | *cyathula orthacantha* |
| Poaceae | *Cynodon nlemfuensis* |
| Cyperaceae | *Cyperus tomaiophyllus* |
| Vitaceae | *Cyphostemma kilimandscharicum* |
| Poaceae | *Dactyloctenium aegyptium* |
| Solanaceae | *Datura suaveolens* |
| Fabaceae | *Desmodium incanum* |
| Fabaceae | *Desmodium intortum* |
| Fabaceae | *Desmodium sp.1* |
| Fabaceae | *Desmodium uncinatum* |
| Acanthaceae | *Dicliptera paniculata* |
| Amaranthaceae | *Digera muricata* |
| Acanthaceae | *Dyschoriste clinopodioides* |
| Acanthaceae | *Dyschoriste hildebrandtii* |
| Asteraceae | *Emilia discifolia* |
| Fabaceae | *Entada leptostachya* |
| Poaceae | *Eragrostis superba* |
| Rosaceae | *Eriobotrya japonica* |
| Fabaceae | *Eriosema sp.1* |
| Ebenaceae | *Euclea racemosa* |
| Euphorbiaceae | *Euphorbia bicompacta* |
| Euphorbiaceae | *Euphorbia cuneata* |
| Euphorbiaceae | *Euphorbia heterophylla* |
| Euphorbiaceae | *Euphorbia hirta* |
| Asteraceae | *Euryops chrysanthemoides* |
| Asteraceae | *Galinsoga parviflora* |
| Asteraceae | *Galinsoga quadriradiata* |
| Geraniaceae | *Geranium vagans* |
| Proteaceae | *Grevillea robusta* |
| Tiliaceae | *Grewia kakothamnos* |
| Tiliaceae | *Grewia tephrodermis* |
| Tiliaceae | *Grewia villosa* |
| Asteraceae | *Gutenbergia cordifolia* |
| Poaceae | *Harpachne schimperi* |
| Asteraceae | *Helichrysum forskahlii* |
| Asteraceae | *Helichrysum glumaceum* |
| Asteraceae | *Helichrysum odoratissimum* |
| Asteraceae | *Helichrysum schimperi* |
| Boraginaceae | *Heliotropium steudneri* |
| Sterculiaceae | *Hermannia exappendiculata* |
| Sterculiaceae | *Hermannia oliveri* |
| Convolvulaceae | *Hewittia malabarica* |
| Malvaceae | *Hibiscus acetosella* |
| Malvaceae | *Hibiscus kabuyeana* |
| Malvaceae | *Hibiscus micranthus* |
| Malvaceae | *Hibiscus sidiformis* |
| Hypericaceae | *Hypericum revolutum* |
| Acanthaceae | *Hypoestes forskaolii* |
| Hypoxidaceae | *Hypoxis obtusa* |
| Lamiaceae | *Hyptis suaveolens* |
| Acanthaceae | *Idiospermum kilimandscharica* |
| Balsaminaceae | *Impatiens hoehnelii* |
| Fabaceae | *Indigofera arrecta* |
| Fabaceae | *Indigofera colutea* |
| Fabaceae | *Indigofera schimperi* |
| Fabaceae | *Indigofera spicata* |
| Fabaceae | *Indigofera tinctoria* |
| Fabaceae | *Indigofera vohemarensis* |
| Convolvulaceae | *Ipomoea batatas* |
| Convolvulaceae | *Ipomoea mombassana* |
| Convolvulaceae | *Ipomoea sp. 1* |
| Acanthaceae | *Justicia calyculata* |
| Acanthaceae | *Justicia debilis* |
| Acanthaceae | *Justicia diclipteroides* |
| Acanthaceae | *Justicia flava* |
| Acanthaceae | *Justicia striata* |
| Crassulaceae | *Kalanchoe lateritia* |
| Asteraceae | *Kleinia squarrosa* |
| Cyperaceae | *Kyllinga brevifolia* |
| Cyperaceae | *Kyllinga bulbosa* |
| Cyperaceae | *Kyllinga sp.1* |
| Lamiaceae | *Lantana camara* |
| Fabaceae | *Leucaena leucocephala* |
| Lamiaceae | *Leucas glabrata* |
| Lamiaceae | *Leucas grandis* |
| Lamiaceae | *Leucas neuflizeana* |
| Lamiaceae | *Lippia javanica* |
| Verbenaceae | *Lippia kituiensis* |
| Lobeliaceae | *Lobelia fervens* |
| Capparaceae | *Maerua endlichii* |
| Capparaceae | *Maerua sp.1* |
| Capparaceae | *Maerua triphylla* |
| Asteraceae | *Melanthera scandens* |
| Malvaceae | *Melhania ovata* |
| Poaceae | *Melinis repens* |
| Fabaceae | *Mimosa pudica* |
| NA | *Moligono sp.1* |
| Commelinaceae | *Murdannia simplex* |
| Fabaceae | *Neonotonia wightii* |
| Solanaceae | *Nicandra physaloides* |
| Lamiaceae | *Ocimum americanum* |
| Lamiaceae | *Ocimum gratissimum* |
| Lamiaceae | *Ocimum Kenyense* |
| Lamiaceae | *Ocimum kilimandscharicum* |
| Asteraceae | *Ocimum sp. 1* |
| Rubiaceae | *Oldenlandia friesiorum* |
| Cucurbitaceae | *Oreosyce africana* |
| Hyacinthaceae | *Ornithogalum tenuifolium* |
| Asteraceae | *Osteospermum vaillantii* |
| Oxalidaceae | *Oxalis corniculata* |
| Polygonaceae | *Oxygonum sinuatum* |
| Rubiaceae | *Paederia pospischilii* |
| Poaceae | *Panicum maximum* |
| Poaceae | *Panicum trichocladum* |
| Fabaceae | *Parochetus communis* |
| Asteraceae | *Parthenium hysterophorus* |
| Passifloraceae | *Passiflora subpeltata* |
| Malvaceae | *Pavonia burchellii* |
| Malvaceae | *Pavonia urens* |
| Pedaliaceae | *Pedaliodiscus macrocarpus* |
| Rubiaceae | *Pentas parvifolia* |
| Rubiaceae | *Pentas zanzibarica* |
| Lauraceae | *Persea sp. 1* |
| Polygonaceae | *Persicaria senegalensis* |
| Polygonaceae | *Persicaria setosula* |
| Euphorbiaceae | *Phyllanthus sp.1* |
| Pittosporaceae | *Pittosporum viridiflorum* |
| NA | *Plant sp. 1* |
| NA | *Plant sp. 2* |
| NA | *Plant sp. 3* |
| NA | *Plant sp. 4* |
| NA | *Plant sp. 5* |
| NA | *Plant sp. 6* |
| NA | *Plant sp. 7* |
| NA | *Plant sp. 8* |
| NA | *Plant sp. 9* |
| NA | *Plant sp. 10* |
| NA | *Plant sp. 11* |
| NA | *Plant sp. 12* |
| NA | *Plant sp. 13* |
| NA | *Plant sp. 14* |
| NA | *Plant sp. 15* |
| NA | *Plant sp. 16* |
| NA | *Plant sp. 17* |
| NA | *Plant sp. 18* |
| NA | *Plant sp. 19* |
| NA | *Plant sp. 20* |
| NA | *Plant sp. 21* |
| NA | *Plant sp. 22* |
| NA | *Plant sp. 23* |
| NA | *Plant sp. 24* |
| NA | *Plant sp. 25* |
| NA | *Plant sp. 26* |
| NA | *Plant sp. 27* |
| NA | *Plant sp. 28* |
| NA | *Plant sp. 29* |
| NA | *Plant sp. 30* |
| NA | *Plant sp. 31* |
| NA | *Plant sp. 32* |
| NA | *Plant sp. 33* |
| NA | *Plant sp. 34* |
| NA | *Plant sp. 35* |
| Lamiaceae | *Platostoma africana* |
| Lamiaceae | *Platostoma denticulatum* |
| Lamiaceae | *Plectranthus alboviolaceus* |
| Lamiaceae | *Plectranthus alpinus* |
| Lamiaceae | *Plectranthus barbatus* |
| Lamiaceae | *Plectranthus caninus* |
| Lamiaceae | *Plectranthus ignarius* |
| Lamiaceae | *Plectranthus kamerunensis* |
| Lamiaceae | *Plectranthus luteus* |
| Lamiaceae | *Plectranthus olostegioides* |
| Lamiaceae | *Plectranthus punctatus* |
| Lamiaceae | *Plectranthus sp.1* |
| polygalaceae | *Polygala sphenoptera* |
| Lamiaceae | *Premna oligocephala* |
| Myrtaceae | *Psidium guajava* |
| Rubiaceae | *Psychotria capensis* |
| Rubiaceae | *Psychotria mahonii* |
| Dennstaedtiaceae | *Pteridium aquilinum* |
| Amaranthaceae | *Pupalia lappacea* |
| Lamiaceae | *Pycnostachys meyeri* |
| Lamiaceae | *Pycnostachys sp.1* |
| Ranunculaceae | *Ranunculus multifidus* |
| Anacardiaceae | *Rhus natalensis* |
| Fabaceae | *Rhynchosia elegans* |
| Fabaceae | *Rhynchosia sp. 1* |
| Rubiaceae | *Richardia brasiliensis* |
| Euphorbiaceae | *Ricinus communis* |
| Lamiaceae | *Rotheca sp. 1* |
| Rosaceae | *Rubus niveus* |
| Rosaceae | *Rubus rosifolius* |
| Rosaceae | *Rubus steudneri* |
| Lamiaceae | *Salvia nilotica* |
| Rhamnaceae | *Scutia myrtina* |
| Anacardiaceae | *Searsia natalensis* |
| Anacardiaceae | *Searsia sp.1* |
| Asteraceae | *Senecio madagascariensis* |
| Asteraceae | *Senecio subsessilis* |
| Asteraceae | *Senecio syringifolius* |
| Fabaceae | *Senna didymobotrya* |
| Fabaceae | *Senna longiracemosa* |
| Fabaceae | *Senna occidentalis* |
| Pedaliaceae | *Sesamum angolense* |
| Fabaceae | *Sesbania sesban* |
| Malvaceae | *Sida acuta* |
| Malvaceae | *Sida alba* |
| Malvaceae | *Sida ovata* |
| Malvaceae | *Sida rhombifolia* |
| Malvaceae | *Sida tenuicarpa* |
| Asteraceae | *Sigesbeckia orientalis* |
| Solanaceae | *Solanum campylacanthum* |
| Solanaceae | *Solanum mauritianum* |
| Solanaceae | *Solanum nigrum* |
| Solanaceae | *Solanum renschii* |
| Asteraceae | *Sonchus luxurians* |
| Asteraceae | *Sonchus oleraceus* |
| Rubiaceae | *Spermacoce princeae* |
| Rubiaceae | *Spermacoce pusilla* |
| Asteraceae | *Sphaeranthus suaveolens* |
| Asteraceae | *Sphagneticola trilobata* |
| Poaceae | *Sporobolus pyramidalis* |
| Verbenaceae | *Stachytarpheta urticifolia* |
| Fabaceae | *Stylosanthes fruticosa* |
| Myrtaceae | *Syzium sp. 1* |
| Apocynaceae | *Tabernaemontana sp. 1* |
| Bignoniaceae | *Tecoma stans* |
| Fabaceae | *Tephrosia hildebrandtii* |
| Fabaceae | *Tephrosia uniflora* |
| Fabaceae | *Tephrosia villosa* |
| Acanthaceae | *Thunbergia alata* |
| Capparaceae | *Thylachium thomasii* |
| Lamiaceae | *Tinnea aethiopica* |
| Asteraceae | *Tithonia diversifolia* |
| Rutaceae | *Toddalia asiatica* |
| Zygophyllaceae | *Tribulus terrestris* |
| Boraginaceae | *Trichodesma zeylanica* |
| Asteraceae | *Tridax procumbens* |
| Fabaceae | *Trifolium semipilosum* |
| Tiliaceae | *Triumfetta brachyceras* |
| Tiliaceae | *Triumfetta rhomboidea* |
| Tiliaceae | *Triumfetta sp.1* |
| Meliaceae | *Turraea robusta* |
| Poaceae | *Urochloa Trichopus* |
| Asteraceae | *Vernonia adoensis* |
| Asteraceae | *Vernonia auriculifera* |
| Asteraceae | *Vernonia brachycalyx* |
| Asteraceae | *Vernonia cinerascens* |
| Asteraceae | *Vernonia glabra* |
| Asteraceae | *Vernonia karaguensis* |
| Asteraceae | *Vernonia lasiopus* |
| Asteraceae | *Vernonia sp.1* |
| Asteraceae | *Vernonia usambarensis* |
| Scrophulariaceae | *Veronica abyssinica* |
| Campanulaceae | *Wahlenbergia abyssinica* |
| Asteraceae | *Waltheria indica* |
| Cucurbitaceae | *Zehneria scabra* |
| Fabaceae | *Zornia setosa* |

**Table S5:** Bee species list

| **Family** | **Species** |
| --- | --- |
| Andrenidae | *Andrena sp. 1* |
| Megachilidae | *Afranthidium concolor* |
| Afromelecta | *Afromelecta sp. 1* |
| Apidae | *Amegilla sp. 1* |
| Apidae | *Amegillasp. 2* |
| Apidae | *Amegilla sp. 3* |
| Apidae | *Amegilla sp. 4* |
| Apidae | *Amegilla sp. 5* |
| Apidae | *Amegilla sp. 6* |
| Apidae | *Amegilla sp. 7* |
| Apidae | *Amegilla sp. 8* |
| Apidae | *Amegilla sp. 9* |
| Apidae | *Amegilla sp. 10* |
| Apidae | *Amegilla sp. 11* |
| Apidae | *Amegilla sp. 12* |
| Apidae | *Amegilla sp. 13* |
| Apidae | *Amegilla sp. 14* |
| Apidae | *Amegilla sp. 15* |
| Apidae | *Amegilla sp. 16* |
| Apidae | *Amegilla sp. 16a* |
| Apidae | *Amegilla sp. 16b* |
| Apidae | *Amegilla sp. 17* |
| Apidae | *Amegilla sp. 18* |
| Apidae | *Amegilla sp. 19* |
| Apidae | *Apis mellifera* |
| Megachilidae | *Anthidium sp. 1* |
| Megachilidae | *Anthidium sp. 2* |
| Apidae | *Anthophora sp. 1* |
| Apidae | *Braunsapissp. 1* |
| Apidae | *Braunsapis sp. 1a* |
| Apidae | *Braunsapis sp. 1b* |
| Apidae | *Braunsapis sp. 2* |
| Apidae | *Braunsapis sp. 3* |
| Apidae | *Braunsapis sp. 4* |
| Apidae | *Braunsapis sp. 5* |
| Apidae | *Braunsapis sp. 6* |
| Apidae | *Braunsapis sp. 7* |
| Apidae | *Ceratina sp. 1* |
| Apidae | *Ceratina sp. 1a* |
| Apidae | *Ceratina sp. 2* |
| Apidae | *Ceratina sp. 3* |
| Apidae | *Ceratina sp. 4* |
| Apidae | *Ceratina sp. 5* |
| Apidae | *Ceratina sp. 6* |
| Apidae | *Ceratina sp. 7* |
| Apidae | *Ceratina sp. 8* |
| Apidae | *Ceratina sp. 9* |
| Apidae | *Ceratina sp. 10* |
| Apidae | *Ceratina sp. 11* |
| Apidae | *Ceratina sp. 12* |
| Apidae | *Ceratina sp. 12a* |
| Apidae | *Ceratina sp. 13* |
| Apidae | *Ceratina sp. 14* |
| Apidae | *Ceratina sp. 15* |
| Apidae | *Ceratina sp. 16* |
| Megachilidae | *Coelioxys sp. 1* |
| Megachilidae | *Coelioxys sp. 2* |
| Colletidae | *Colletes sp. 1* |
| Colletidae | *Colletes sp. 2* |
| Colletidae | *Colletes sp. 3* |
| Apidae | *Ctenoplectrina sp. 1* |
| Apidae | *Ctenoplectrina sp. 2* |
| Megachilidae | *Heriades sp. 1* |
| Megachilidae | *Heriades sp. 2* |
| Megachilidae | *Heriades sp. 3* |
| Colletidae | *Hylaeus sp. 1* |
| Colletidae | *Hylaeus sp. 2* |
| Apidae | *Hypotrigona sp. 1* |
| Apidae | *Hypotrigona sp. 2* |
| Halictidae | *Lasioglossum sp. 1* |
| Halictidae | *Lasioglossum sp. 2* |
| Halictidae | *Lasioglossum sp. 2a* |
| Halictidae | *Lasioglossum sp. 3* |
| Halictidae | *Lasioglossum sp. 4* |
| Halictidae | *Lasioglossum sp. 5* |
| Halictidae | *Lasioglossum sp. 6* |
| Halictidae | *Lipotriches sp. 1* |
| Halictidae | *Lipotriches sp. 1a* |
| Halictidae | *Lipotriches sp. 2* |
| Halictidae | *Lipotriches sp. 3* |
| Halictidae | *Lipotriches sp. 4* |
| Halictidae | *Lipotriches sp. 5* |
| Halictidae | *Lipotriches sp. 6* |
| Halictidae | *Lipotriches sp. 7* |
| Halictidae | *Lipotriches sp. 8* |
| Halictidae | *Lipotriches sp. 9* |
| Halictidae | *Lipotriches sp. 10* |
| Halictidae | *Lipotriches sp. 11* |
| Halictidae | *Lipotriches sp. 12* |
| Halictidae | *Lipotriches sp. 13* |
| Halictidae | *Lipotriches sp. 14* |
| Halictidae | *Lipotriches sp. 15* |
| Halictidae | *Lipotriches sp. 16* |
| Halictidae | *Lipotriches sp. 17* |
| Halictidae | *Lipotriches sp. 18* |
| Apidae | *Macrogalea candida* |
| Apidae | *Macrogalea sp. 2* |
| Megachilidae | *Megachile ferina* |
| Megachilidae | *Megachile sp. 1* |
| Megachilidae | *Megachile sp. 1a* |
| Megachilidae | *Megachile sp. 1b* |
| Megachilidae | *Megachile sp. 1c* |
| Megachilidae | *Megachile sp. 2* |
| Megachilidae | *Megachile sp. 3* |
| Megachilidae | *Megachile sp. 4* |
| Megachilidae | *Megachile sp. 4a* |
| Megachilidae | *Megachile sp. 5* |
| Megachilidae | *Megachile sp. 5a* |
| Megachilidae | *Megachile sp. 6* |
| Megachilidae | *Megachile sp. 7* |
| Megachilidae | *Megachile sp. 8* |
| Megachilidae | *Megachile sp. 9* |
| Megachilidae | *Megachile sp.10* |
| Megachilidae | *Megachile sp. 11* |
| Megachilidae | *Megachile sp. 12* |
| Megachilidae | *Megachile sp. 13* |
| Megachilidae | *Megachile sp. 14* |
| Megachilidae | *Megachile sp. 14a* |
| Megachilidae | *Megachile sp. 15* |
| Megachilidae | *Megachile sp. 16* |
| Megachilidae | *Megachile sp. 17* |
| Megachilidae | *Megachile sp. 18* |
| Megachilidae | *Megachile sp.19* |
| Apidae | *Melliponula sp. 1* |
| Apidae | *Meliponine sp. 2* |
| Halictidae | *Nomia sp. 1* |
| Halictidae | *Nomia sp. 2* |
| Apidae | *Pacymelus sp. 1* |
| Apidae | *Cleptoparasitic sp. 1* |
| Apidae | *Cleptoparasitic sp. 2* |
| Apidae | *Cleptoparasitic sp. 3* |
| Apidae | *Cleptoparasitic sp. 4* |
| Apidae | *Cleptoparasitic sp. 5* |
| Halictidae | *Patellapis sp. 1* |
| Halictidae | *Patellapis sp. 2* |
| Halictidae | *Patellapis sp. 3* |
| Halictidae | *Patellapis sp. 4* |
| Halictidae | *Patellapis sp. 5* |
| Halictidae | *Patellapis sp. 6* |
| Halictidae | *Patellapis sp. 7* |
| Halictidae | *Patellapis sp. 8* |
| Halictidae | *Patellapis sp. 9* |
| Apidae | *Plebeina armata* |
| Apidae | *Plebeina sp. 1* |
| Halictidae | *Pseudapis sp. 1* |
| Halictidae | *Pseudapis sp. 1a* |
| Halictidae | *Pseudapis sp. 2* |
| Halictidae | *Pseudapis sp. 3* |
| Halictidae | *Pseudapis sp. 4* |
| Halictidae | *Pseudapis sp. 5* |
| Halictidae | *Pseudapis sp. 6* |
| Halictidae | *Pseudapis sp. 7* |
| Megachilidae | *Pseudoanthidium sp. 1* |
| Megachilidae | *Pseudoanthidium sp. 2* |
| Megachilidae | *Pseudoanthidium sp. 3* |
| Megachilidae | *Pseudoanthidium sp. 4* |
| Megachilidae | *Pseudoanthidium sp. 5* |
| Megachilidae | *Pseudoanthidium sp. 6* |
| Halictidae | *Seladonia sp. 1* |
| Halictidae | *Seladonia sp. 2* |
| Halictidae | *Seladonia sp. 3* |
| Halictidae | *Seladonia sp. 4* |
| Halictidae | *Seladonia sp. 5* |
| Halictidae | *Sphecodes sp. 1* |
| Halictidae | *Sphecodes sp. 2* |
| Halictidae | *Steganomus sp. 1* |
| Halictidae | *Steganomus sp. 2* |
| Apidae | *Tetralonia sp. 1* |
| Apidae | *Tetralonia sp. 2* |
| Apidae | *Tetraloniella sp. 1* |
| Apidae | *Tetraloniella sp. 2* |
| Apidae | *Tetraloniella sp. 3* |
| Apidae | *Tetraloniella sp. 4* |
| Halictidae | *Thrincostoma sp. 1* |
| Apidae | *Thyreus sp. 1 (pictus)* |
| Apidae | *Thyreus sp. 2* |
| Apidae | *Xylocopa caffra* |
| Apidae | *Xylocopa calens* |
| Apidae | *Xylocopa flavicolis* |
| Apidae | *Xylocopa flavorufa* |
| Apidae | *Xylocopa inconstans* |
| Apidae | *Xylocopa nigrita* |
| Apidae | *Xylocopa sp. 1* |
| Apidae | *Xylocopa sp. 2* |
| Apidae | *Xylocopa sp. 3* |
| Apidae | *Xylocopa sp. 4* |


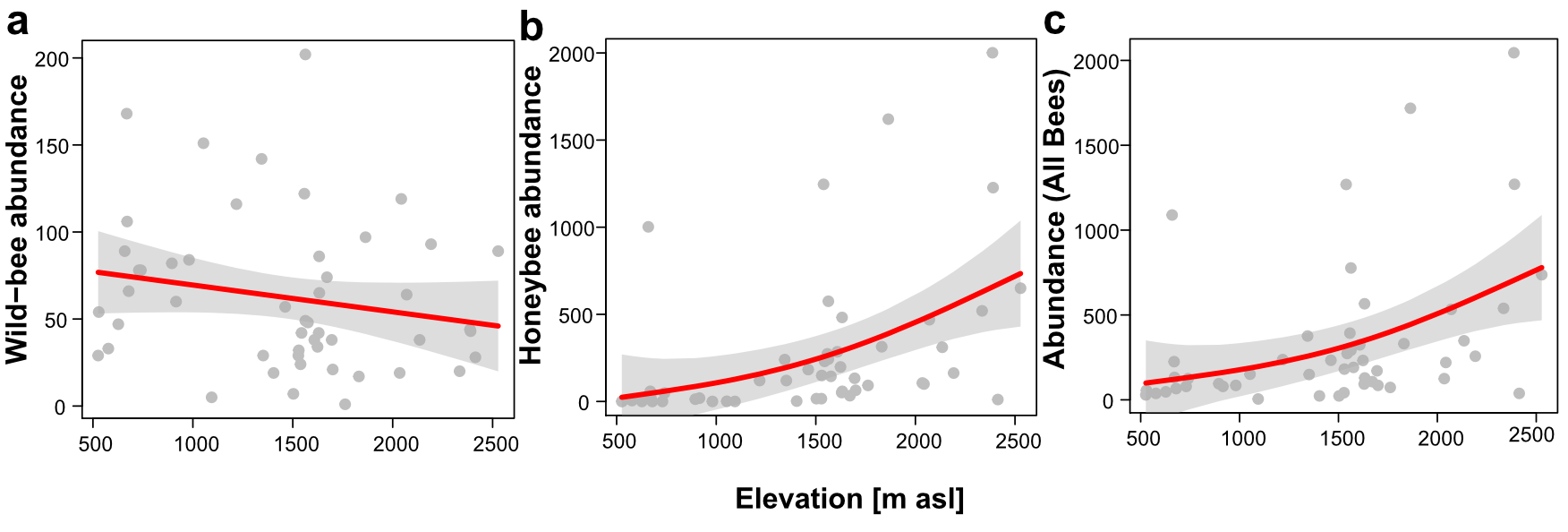


**Figure S1:** Elevational patterns of bee abundance. a= wild bee abundance did not show any pattern with increasing elevation (n= 50, Explained deviance (ED) = 3.87%, p = 0.17), b= honeybee abundance increased significantly with increasing elevation (n=50, ED = 21.8%, p = 0.01), and c= combined abundance of honeybees and wild bees also increased with increasing elevation (n= 50, ED =19.7%, p =0.01). All abundance patterns were analysed using generalized additive models (Gaussian family, basis dimension *k* = 5). Red trend lines with circular dots represent observed bee abundance per study plot.


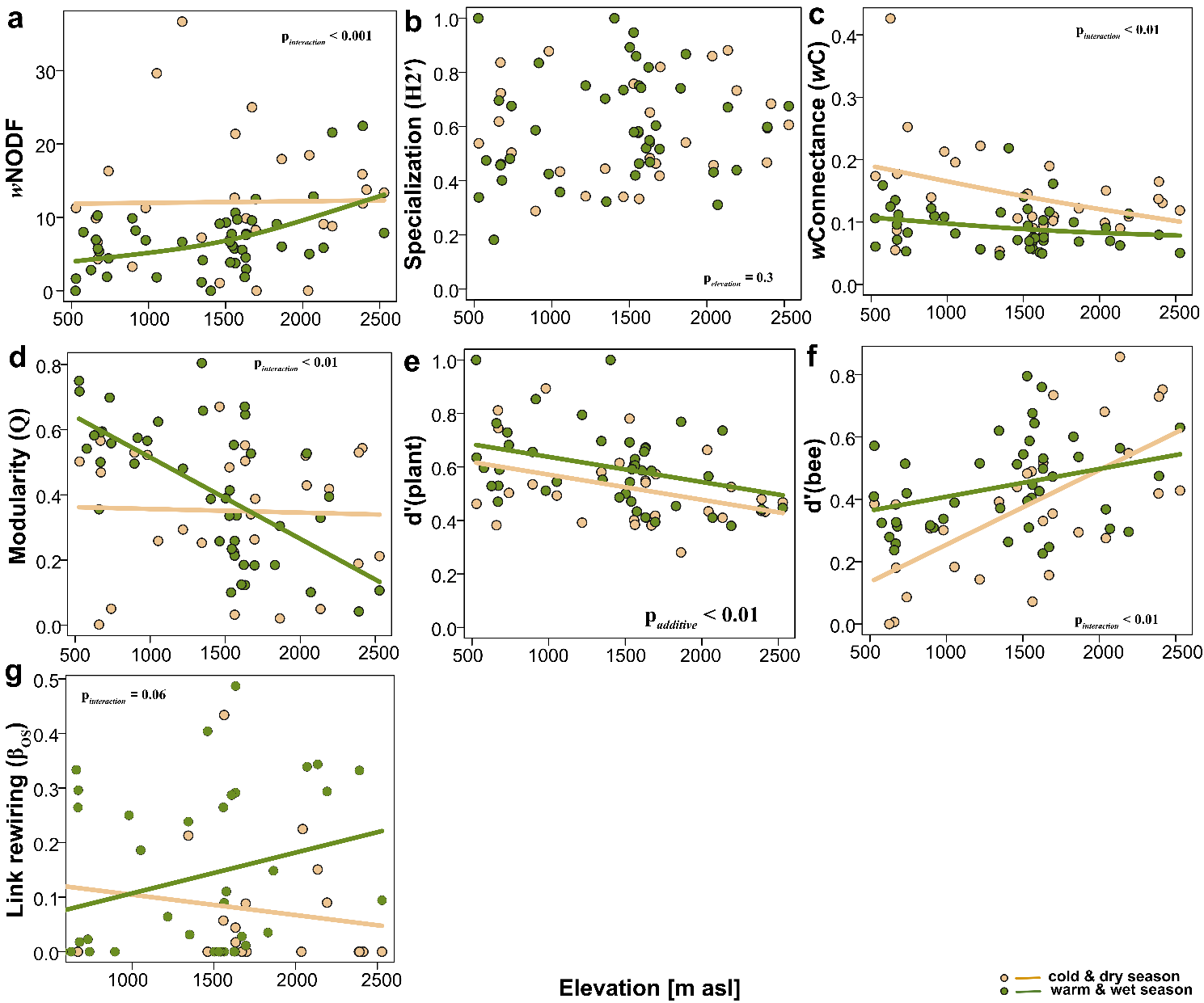


**Figure S2:** Seasonal and elevational patterns of bee-plant interaction network indices after correcting for network size effect (removing interactions with less than 10 nodes). (a) *w*NODF: weighted nestedness, (b) H2': network specialisation, (c) *w*C: weighted connectance, (d) Q: modularity, (e) d'plant: plant species specialisation, (f) d'bee: bee species specialisation, (g) β_OS_: link rewiring). All seasonal network trends were analysed using generalized additive models (Gaussian family, basis dimension *k* = 5). The p-values within boxes indicate the statistical differences for each network index between the two seasons across elevation (i.e., cold-dry and warm-wet).





**Figure S3:** Plant-bee interactions during the cold-dry season. Bee pollinator species (yellow vertical and horizontal bars at the top) recorded visiting plant species (green vertical bars at the bottom). Grey lines indicates links between bee and plant species. The width of each vertical bar indicates visitation frequency of each species, while the size of the lines indicates interaction strength of each species.


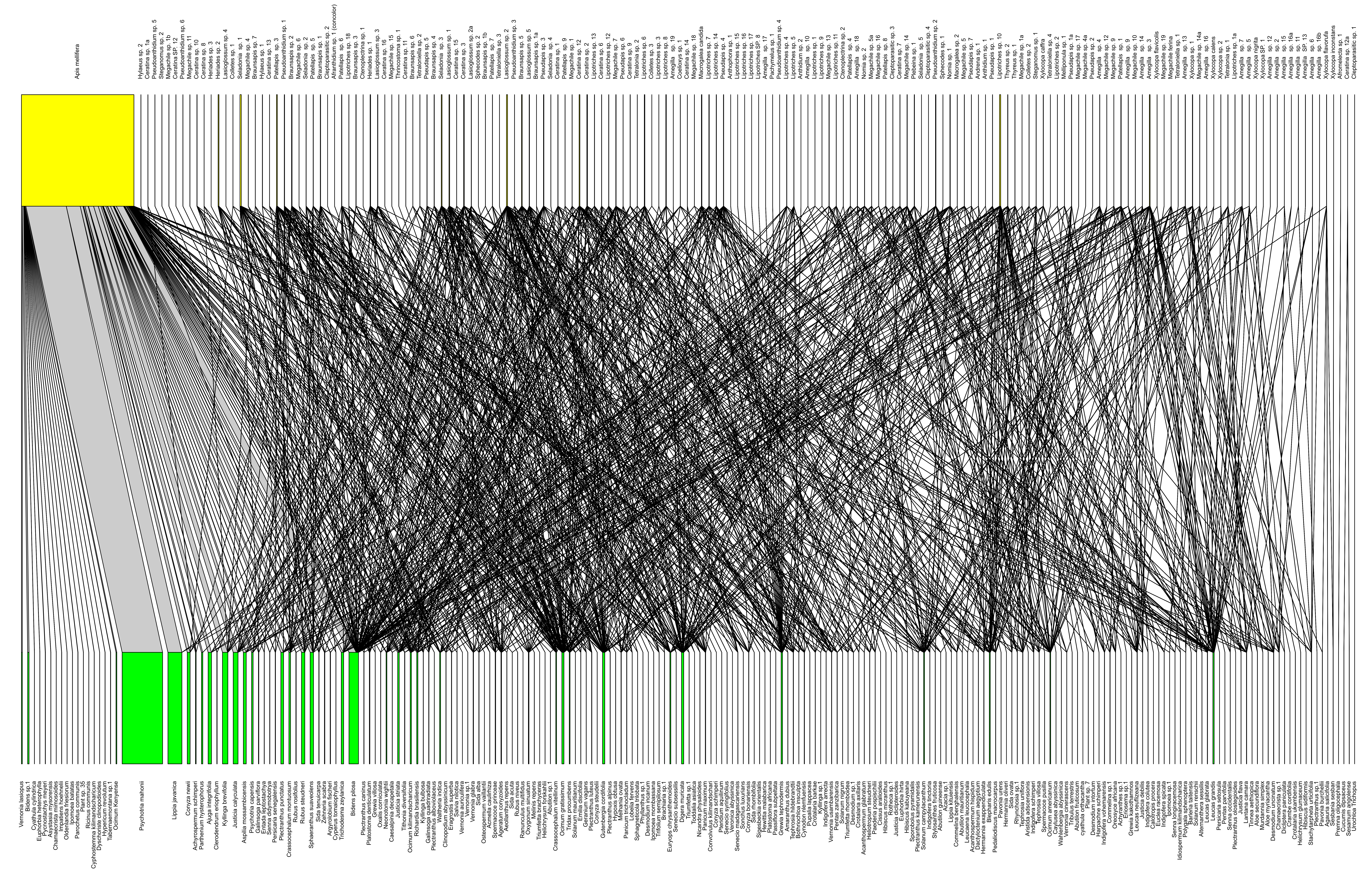


**Figure S4:** Plant-bee interactions during the warm-wet season. Bee pollinator species (yellow vertical bars at the top) recorded visiting plant species (green vertical bars at the bottom). Grey lines indicates links between bee and plant species. The width of each vertical bar indicates visitation frequency of each species, while the size of the lines indicates interaction strength of each species.

**Table S6:** Lists of all standardized explanatory variables (standardized beta) of best fit models (ΔAIC_c_ < 3) for explaining seasonal interaction patterns (*w*NODF: weighted nestedness, H2': network specialisation, Q: modularity, *w*C: weighted connectance, d'bee: bee species specialisation, d'plant: plant species specialisation, β_OS_: link rewiring). Log transformed bee abundance (log(abun)), mean monthly precipitation (MMP), Mean monthly temperature (MMT), plant species richness (F_γ_), and bee species richness (b_γ_). Models with smallest AIC_c_ and **Δ**AIC_c_ denotes the best models, allowing a direct evaluation of effect strengths among the explanatory variables.

|  | **b_γ_** | **log(abn)** | **MMP** | **MMT** | **F_γ_** | **R^2^** | **AICc** | **ΔAIC_c_** | **weight** |
| --- | --- | --- | --- | --- | --- | --- | --- | --- | --- |
|  |  |  |  | -0.26* | -0.20* | 0.12 | 744.9 | 0 | 0.188 |
|  | 0.26 |  |  | -0.35** | -0.39* | 0.13 | 745.1 | 0.25 | 0.167 |
| *w*NODF |  |  |  | -0.28** |  | 0.08 | 746.7 | 1.83 | 0.075 |
|  | 0.29 | -0.07 |  | -0.38** | -0.38* | 0.14 | 747 | 2.12 | 0.065 |
|  |  |  | -0.04 | -0.26* | -0.18 | 0.12 | 747 | 2.13 | 0.065 |
|  |  | -0.02 |  | -0.27* | -0.19 | 0.12 | 747.1 | 2.19 | 0.063 |
|  | 0.26 |  | -0.05 | -0.36** | -0.37* | 0.14 | 747.2 | 2.37 | 0.058 |
|  |  |  | -0.13 | -0.29** |  | 0.09 | 747.3 | 2.4 | 0.057 |
|  |  | -0.11 |  | -0.30** |  | 0.09 | 747.8 | 2.94 | 0.043 |
|  | -0.59*** | 0.46*** |  | 0.26* |  | 0.28 | -12.1 | 0 | 0.365 |
| H2' | -0.64*** | 0.46*** | 0.13 | 0.29* |  | 0.29 | -11.5 | 0.62 | 0.269 |
|  | -0.67*** | 0.46*** |  | 0.28* | 0.09 | 0.28 | -10.2 | 1.91 | 0.141 |
|  | -0.68*** | 0.46*** | 0.12 | 0.30* | 0.04 | 0.29 | -9.2 | 2.89 | 0.086 |
|  | 0.30* | -0.73*** |  | 0.20* | 0.32** | 0.68 | 60.965 | 0 | 0.585 |
| Q | 0.30* | -0.73*** | 0 | 0.20* | 0.32** | 0.68 | -106.6 | 2.34 | 0.182 |
|  | -0.16 | -0.28*** |  |  | -0.45*** | 0.57 | -239 | 0 | 0.234 |
|  |  | -0.30*** |  |  | -0.57*** | 0.56 | -238.9 | 0.08 | 0.225 |
|  |  | -0.32*** |  | -0.09 | -0.55*** | 0.57 | -238.4 | 0.64 | 0.17 |
| *w*C | -0.17 | -0.28*** | -0.05 |  | -0.42*** | 0.57 | -237.1 | 1.87 | 0.092 |
|  | -0.12 | -0.30*** |  | -0.04 | -0.47*** | 0.57 | -237 | 2.04 | 0.084 |
|  |  | -0.29*** | -0.04 |  | -0.55*** | 0.56 | -237 | 2.04 | 0.084 |
|  |  | -0.32*** | -0.06 | -0.1 | -0.52*** | 0.57 | -236.7 | 2.35 | 0.072 |
|  | -0.83*** | 0.23* |  |  | 0.72*** | 0.35 | -22.6 | 0 | 0.406 |
| d'bee | -0.82*** | 0.22* | 0.11 |  | 0.67*** | 0.36 | -21.5 | 1.11 | 0.234 |
|  | -0.86*** | 0.24* |  | 0.03 | 0.74*** | 0.35 | -20.4 | 2.22 | 0.134 |
|  |  |  | 0.17 | 0.31** |  | 0.12 | -18.7 | 0 | 0.161 |
|  |  |  |  | 0.29** |  | 0.09 | -17.9 | 0.74 | 0.111 |
|  | 0.16 |  |  | 0.23* |  | 0.11 | -17.9 | 0.77 | 0.109 |
| d'plant | 0.1 |  | 0.13 | 0.27* |  | 0.12 | -17.1 | 1.54 | 0.074 |
|  |  |  |  | 0.29** | 0.11 | 0.1 | -17 | 1.67 | 0.07 |
|  |  |  | 0.15 | 0.31** | 0.04 | 0.12 | -16.6 | 2.13 | 0.055 |
|  |  | 0.02 | 0.17 | 0.32** |  | 0.12 | -16.5 | 2.21 | 0.053 |
|  |  | 0.06 |  | 0.31** |  | 0.09 | -16.1 | 2.59 | 0.044 |
|  | 0.25* |  |  |  |  | 0.06 | -15.7 | 2.98 | 0.036 |
|  | 0.17 | -0.01 |  | 0.22 |  | 0.11 | -15.7 | 2.99 | 0.036 |
|  | 0.33** | 0.26* |  |  |  | 0.22 | -85.6 | 0 | 0.209 |
|  | 0.40** | 0.2 |  | -0.13 |  | 0.23 | -84.2 | 1.41 | 0.103 |
| β_OS_ | 0.30* | 0.23 | 0.1 |  |  | 0.23 | -84 | 1.58 | 0.095 |
|  | 0.49*** |  |  | -0.24 |  | 0.2 | -83.9 | 1.68 | 0.09 |
|  | 0.32 | 0.26* |  |  | 0.01 | 0.22 | -83.2 | 2.33 | 0.065 |
|  | 0.44** |  | 0.13 | -0.21 |  | 0.21 | -82.8 | 2.74 | 0.053 |

**References:**

Karger, D. N., Conrad, O., Böhner, J., Kawohl, T., Kreft, H., Soria-Auza, R. W., Zimmermann, N. E., Linder, H. P., & Kessler, M. (2017). Climatologies at high resolution for the earth’s land surface areas. *Scientific Data*, *4*, 1–20. <https://doi.org/10.1038/sdata.2017.122>

Brun, P., Zimmermann, N. E., Hari, C., Pellissier, L., and Karger, D. N.: Global climate-

related predictors at kilometre resolution for the past and future, Earth Syst. Sci. Data

Discuss. [preprint], https://doi.org/10.5194/essd-2022-212, in review, 2022
